# Dietary omega-6 lipids promote postinjury aberrant bone formation in obesity

**DOI:** 10.1172/JCI205829

**Published:** 2026-06-11

**Authors:** Stefanie L. Moye, Monisha Mittal, Tarun Srinivasan, Sneha Korlakunta, Chase A. Pagani, Ayelet Dar, Oromo Geshow, Dylan Feist, Lauren G. Zacharias, Zhao Li, Aaron W. James, Gerta Hoxhaj, Andrew M. Smith, Katherine A. Gallagher, Thomas P. Mathews, Robert J. Tower, Benjamin Levi

**Affiliations:** 1 Department of Surgery,; 2 Medical Scientist Training Program,; 3 Department of Immunology, and; 4 Children’s Medical Center Research Institute, University of Texas Southwestern Medical Center, Dallas, Texas, USA.; 5 Department of Pathology, Johns Hopkins School of Medicine, Baltimore, Maryland, USA.; 6 Department of Bioengineering, University of Illinois Urbana-Champaign, Urbana, Illinois, USA.; 7 Department of Surgery and; 8 Department of Microbiology, University of Michigan School of Medicine, Ann Arbor, Michigan, USA.

**Keywords:** Bone biology, Inflammation, Metabolism, Bone disease, Innate immunity, Obesity

## Abstract

Obesity is associated with impaired wound healing, but the mechanisms linking excess adiposity to aberrant tissue repair remain unresolved. Heterotopic ossification (HO) is a severe example of pathologic tissue repair in which mesenchymal progenitor cells (MPCs) undergo aberrant osteochondral differentiation within soft tissue, leading to joint contractures and pain. Here, we show that accumulation of dietary omega-6 (ω-6) lipids in the injury site is a key mechanism linking obesity to HO. Specifically, in mice fed a high-fat diet (HFD), injured tissues were enriched in linoleic and arachidonic acids, providing substrate for myeloid COX-2–dependent prostaglandin E_2_ (PGE_2_) production. PGE_2_ then drove a transcriptional program in MPCs that promoted osteochondral differentiation. An isocaloric, low linoleic acid HFD reduced HO despite comparable obesity, demonstrating that dietary lipid composition, rather than adiposity alone, drove pathological repair. Clinical data mirrored these findings, showing that obesity conferred increased HO risk, and COX-2 inhibition reduced HO exclusively in obese patients. Together, these findings identify injury site ω-6 lipid enrichment as the key signal linking the diet to MPC reprogramming, pointing to dietary lipid modulation as an actionable strategy to limit HO in obesity.

## Abstract 2

After trauma or orthopedic surgery, a high-fat diet alters lipid metabolism at the injury site, amplifying inflammation and promoting aberrant bone formation.

## Graphical abstract

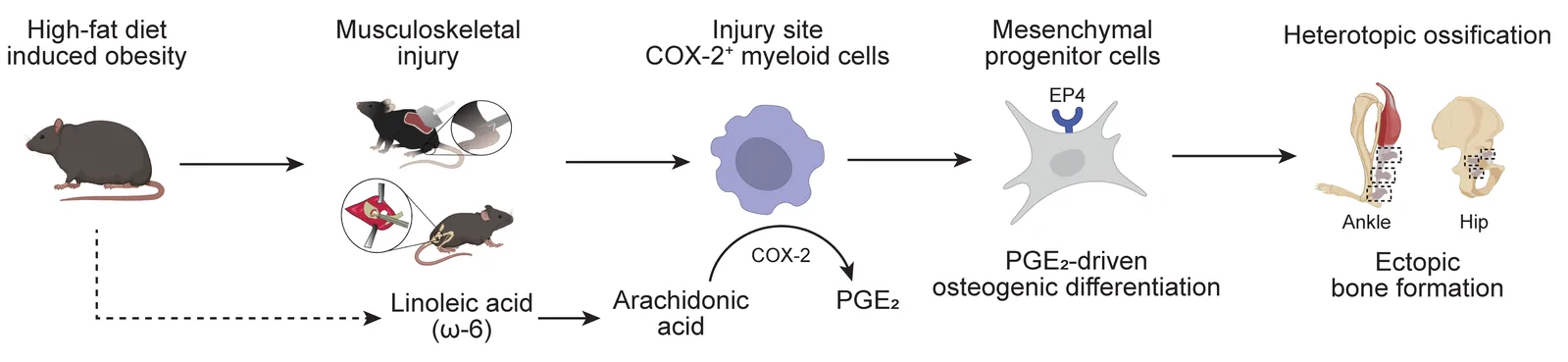

## Introduction

Obesity is a global health crisis affecting over 1 billion individuals worldwide (1). Beyond the well-established associations with cardiovascular and metabolic disease, obesity is increasingly recognized as a critical regulator of tissue injury and repair (2, 3). Obesity impairs wound healing, disrupts fracture repair, and promotes fibrosis and infection, indicating its broad impact on reshaping the cellular and molecular programs that govern tissue regeneration (3–6).

Heterotopic ossification (HO), the pathologic formation of bone in extraskeletal tissues, is a frequent and debilitating consequence of trauma, burn injury, and orthopedic surgery (7). Ectopic bone formation causes pain, joint ankylosis, and functional limitations, often requiring complex surgical intervention, which rarely restores function and often results in recurrence (8). Existing prophylaxes, including NSAIDs and radiation, have inconsistent efficacy, yet the biological basis for this variability remains poorly understood (9–11). While male sex and a prior history of HO are known risk factors, recent retrospective studies suggest that obesity may also increase HO risk (12–15). However, the mechanisms underlying this association are undefined, and it is unknown whether obese individuals require distinct therapeutic approaches to prevent HO.

Obesity is defined by chronic nutrient excess and dysregulated lipid metabolism, which is often amplified by the Western diet (WD), characterized by excessive dietary linoleic acid and a high omega-6/omega-3 (ω-6/ω-3) ratio (16, 17). In obesity, tissues can become enriched with ω-6 polyunsaturated fatty acids (PUFAs), including linoleic acid (LA) and arachidonic acid (AA) (18, 19). These lipids serve as precursors for proinflammatory eicosanoids, which can alter immune signaling and stromal cell differentiation (20, 21). While ω-6 fatty acids and their derivatives have been broadly linked to bone function and inflammatory joint diseases, their role in aberrant bone formation after injury and surgery remains unclear (22–24).

Here, we used traumatic and surgical HO models in the setting of high-fat diet–induced (HFD-induced) obesity to define how dietary lipids affect injury/surgery-site lipid composition, metabolism, and tissue repair. We found that HFD markedly accelerated HO and impaired functional recovery, while amplifying the acute postinjury inflammatory response. Metabolomic and lipidomic profiling revealed substantial enrichment of ω-6 PUFAs, including LA and AA, together with broad lipid class remodeling at the injury site. This accumulation of ω-6 lipids provided substrate for COX-2–dependent prostaglandin E_2_ (PGE_2_) production in infiltrating myeloid cells, promoting osteochondral differentiation of mesenchymal progenitor cells (MPCs) and exacerbating HO formation. Reducing dietary LA or inhibiting myeloid-specific COX-2 signaling in HFD-induced obesity mitigated HO, demonstrating that injury-site ω-6 lipid availability regulates aberrant bone formation. Together, these findings identify injury-site lipid remodeling as a driver of aberrant postinjury bone formation and support modulation of lipid composition as a precision prophylactic strategy for HO in obesity.

## Results

### Obesity enhances HO formation and impairs functional recovery after injury.

**Figure 1:**
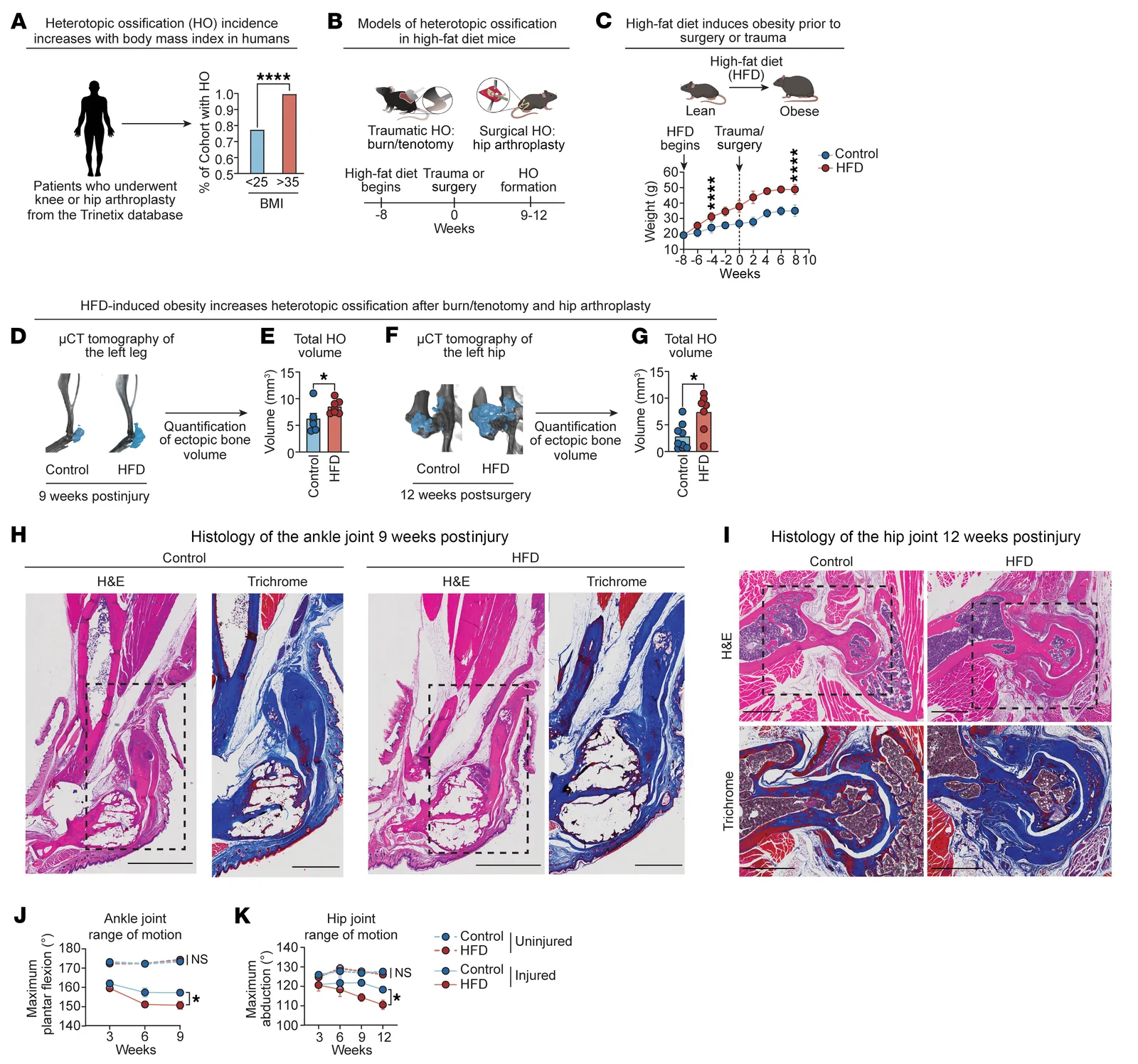
Obesity enhances HO formation and impairs functional recovery after injury. (**A**) TriNetX analysis of HO incidence after total joint arthroplasty in patients considered obese (BMI > 35) versus those with a normal weight (BMI 18.5–24.9) (*n* = 622,009 patients/cohort). (**B**) Experimental design: mice fed an HFD (60% fat) or control diet (10% fat) for 8 weeks prior to B/T or HA, followed for 9–12 weeks before μCT. (**C**) Body weight after diet initiation (*n* = 10–15 mice/group). Surgery performed at week 0. (**D** and **E**) Representative μCT images and quantification of ankle HO in HFD and control mice (9 weeks after B/T; *n* = 6–7 mice/group). (**F** and **G**) Representative μCT images and quantification of hip HO (12 weeks after HA; *n* = 7–9 mice/group). (**H**) Histological images of injured hind limbs (H&E, Masson’s trichrome) at 9 weeks, with outlined regions demonstrating newly formed HO in the calcaneus and Achilles tendon. Scale bars: 2.5 mm (left), 1 mm (right). (**I**) Histological images of injured hips at 12 weeks, with outlined regions demonstrating newly formed HO at the acetabulo-femoral joint. Scale bars: 1 mm. (**J**) Ankle range of motion over time in injured and uninjured limbs (3, 6, and 9 weeks; *n* = 5–12 mice/group; statistical analyses performed at 9 weeks). (**K**) Hip range of motion over time in injured and uninjured hips (3, 6, 9, and 12 weeks; *n* = 5–6 mice/group; statistical analyses performed at 12 weeks). Data represent at least 2 independent experiments. Means ± SEM are plotted. Statistics: χ^2^ (**A**), 2-tailed Student’s *t* test (**C**), or Mann-Whitney test (**E**, **G**, **J**, and **K**). *P < 0.05; ****P < 0.0001. See also Supplemental Figures 1 and 2.

To examine the association between obesity and HO, we analyzed a large retrospective cohort of patients who underwent total joint arthroplasty using the TriNetX database (>110 million US patients). After 1:1 propensity score matching for age, sex, and relevant comorbidities, we compared 622,009 patients with obesity (BMI > 35) and 622,009 nonobese controls (BMI = 18.5–24.9). We found that obesity was associated with a 28% increased risk of postoperative HO (0.997% vs. 0.774%; *P <* 0.0001, risk ratio = 1.289) (Figure 1A). These data extend the conclusions of prior case studies to a large, national patient cohort (13–15).

To test whether diet-induced obesity causally enhances HO, we employed 2 well-established mouse models of traumatic and surgery-associated HO: burn/tenotomy (B/T) and hip arthroplasty (HA) (25–27). Mice were fed an HFD or control diet for 8 weeks prior to injury to induce obesity (Figure 1B and Supplemental Table 1; supplemental material available online with this article; https://doi.org/10.1172/JCI205829DS1). HFD-fed mice exhibited increased body weight, elevated adiposity, and hepatic steatosis without the loss of lean mass (Figure 1C and Supplemental Figure 1, A and C). Consistent with dysregulated metabolism, HFD mice had markedly higher serum leptin and resistin (Supplemental Figure 1B) (28).

After B/T, HFD mice developed significantly greater ectopic bone volume in the injured ankle compared with control-fed mice, as assessed by micro–computed tomography (μCT) at 9 weeks after injury (Figure 1, D and E). In the B/T model, the majority of HO forms around the calcaneus with additional bone formation adjacent to the Achilles tendon (25). Similar increases in HO were observed at the injured hip joint in the HA model after 12 weeks (Figure 1, F and G). In HA, ectopic bone forms in the periacetabular region, including around the subchondral bone (27). Histological analysis (3 weeks after injury) demonstrated increased Safranin O and trichrome staining in HFD mice, reflecting greater cartilage and collagen deposition at the injury site, consistent with increased endochondral ossification (Supplemental Figure 1E) (7). HFD mice also had increased ectopic bone formation at later time points (9 weeks B/T; 12 weeks HA) (Figure 1, H and I; HO regions outlined in black). Additionally, wound healing was markedly delayed with HFD (Supplemental Figure 1D).

Functionally, injured HFD mice exhibited impaired range of motion in the ankle and hip compared with injured controls (Figure 1, J and K). Injured HFD mice also had reduced locomotor activity and rearing, while uninjured mice performed similarly on functional assessments across diets (Supplemental Figure 1, F–H). These deficits were not explained by increased mechanical loading from excess body weight, as independent gait and black box pressure analyses demonstrated similar reductions in loading between HFD and control mice after injury (Supplemental Figure 1, I–K, and Supplemental Videos 1–4) (29, 30). Together, these findings demonstrate that HFD mice experience greater HO and worse functional recovery after injury and that these impairments occur independent of body weight–associated loading.

To test whether the increase in HO is a general feature of diet-induced obesity rather than unique to a single high-fat formulation, we evaluated mice fed a WD, which combines high fat with high sugar to induce obesity (Supplemental Table 1 and Supplemental Figure 2A) (31). WD mice also had increased HO volume compared with controls (B/T; Supplemental Figure 2, B and C). These findings show that distinct obesogenic diets similarly enhance HO, supporting a role for diet-driven metabolic state in predisposing injured tissues to aberrant bone formation.

### HFD-induced obesity amplifies the injury-induced cytokine response at the site of HO formation.

**Figure 2:**
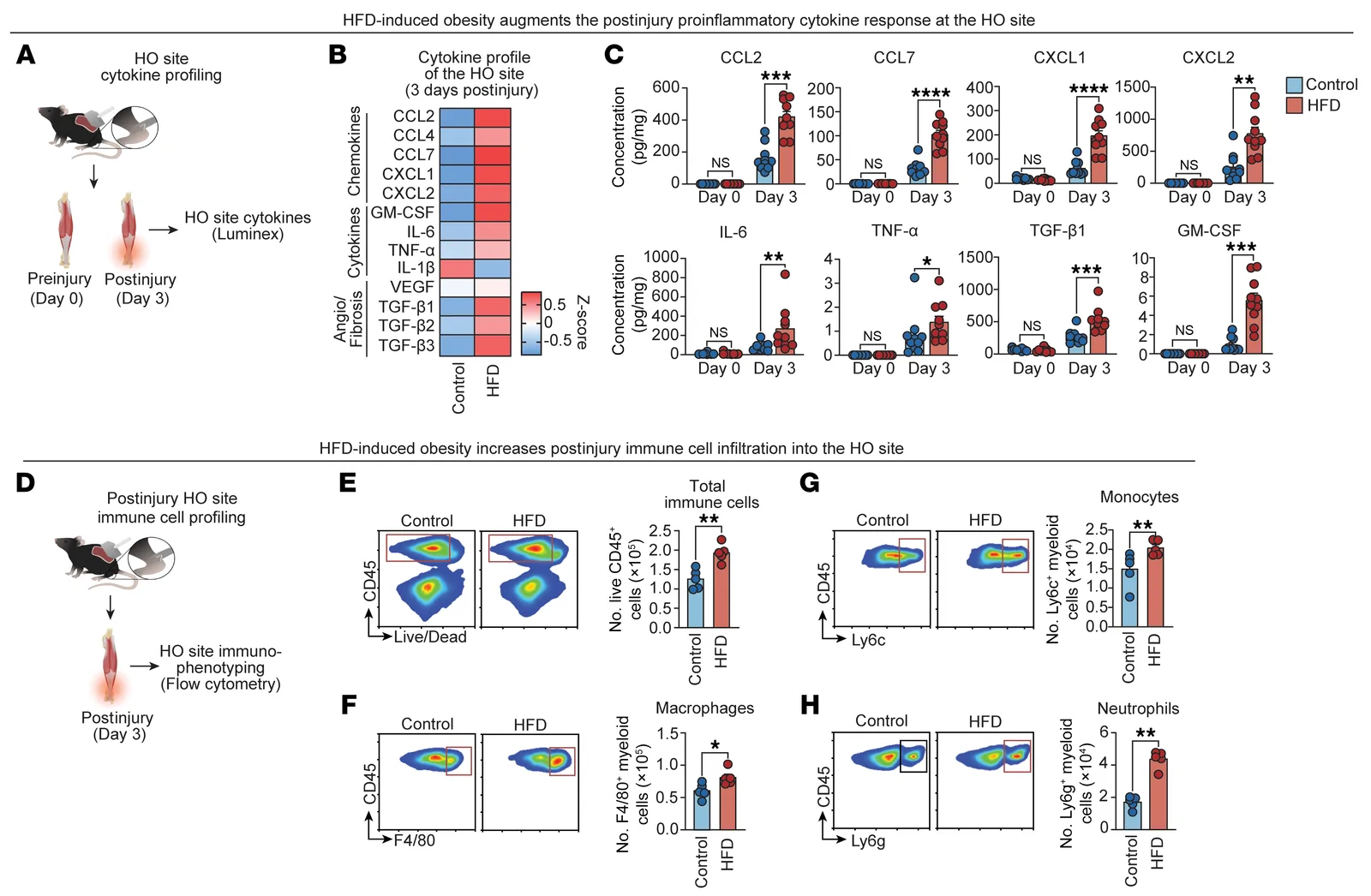
HFD-induced obesity amplifies the injury-induced cytokine response at the site of HO formation. (**A**) Schematic of HO-site cytokine profiling of HFD and control mice (day 0 and day 3 after B/T). (**B**) Heatmap of cytokines/chemokines in the HO site (*z* scores; *n* = 9–10 mice/group). (**C**) Quantification of key cytokines (CCL2, CCL7, CXCL1/2, IL-6, TNF-α, TGF-β1, GM-CSF) normalized to protein (*n* = 9–10 mice/group). (**D**) Schematic of HO-site immune cell profiling (day 3 after injury). (**E**–**H**) Representative flow cytometry plot (left) and quantification (right) of number of live CD45^+^ immune cells, macrophages (F4/80^+^ myeloid cells), monocytes (Ly6c^+^ myeloid cells), and neutrophils (Ly6g^+^ myeloid cells) (*n* = 5 mice/group). Data represent at least 2 independent experiments. Means ± SEM are plotted. Statistics: Mann-Whitney test (**C** and **E**–**H**). *P < 0.05; **P < 0.01; ***P < 0.001; ****P < 0.0001. See also Supplemental Figure 3.

Having established that HFD enhanced HO and worsened functional recovery, we next asked whether obesity alters the early inflammatory response at the HO site. We profiled the cytokine content in lysates isolated from the HO site (injured Achilles tendon and surrounding soft tissue where ectopic bone forms) at baseline (day 0) and day 3 after injury (Figure 2A). Cytokines were nearly undetectable at baseline, whereas by day 3, HFD HO sites displayed substantially higher levels of CCL2, CCL7, CXCL1, CXCL2, IL-6, TNF-α, TGF-β1, and GM-CSF compared with injured controls (Figure 2, B and C). These factors coordinate leukocyte recruitment, monocyte/macrophage activation, and early matrix remodeling (32, 33), indicating that HFD intensifies the local inflammatory response to injury. In contrast, systemic changes were minimal, with only IL-6 elevated in HFD serum after injury (Supplemental Figure 3A).

To further define the composition of the HO microenvironment, we performed flow cytometry at 3 days after injury (Figure 2D). HFD HO sites contained increased numbers of immune cells, including macrophages, monocytes, and neutrophils (Figure 2, E–H, and Supplemental Figure 3B). These results define a heightened inflammatory microenvironment in HFD mice, characterized by amplified cytokine induction and increased recruitment of proinflammatory myeloid cells.

### HFD-induced obesity enriches ω-6 fatty acids at the HO site.

**Figure 3:**
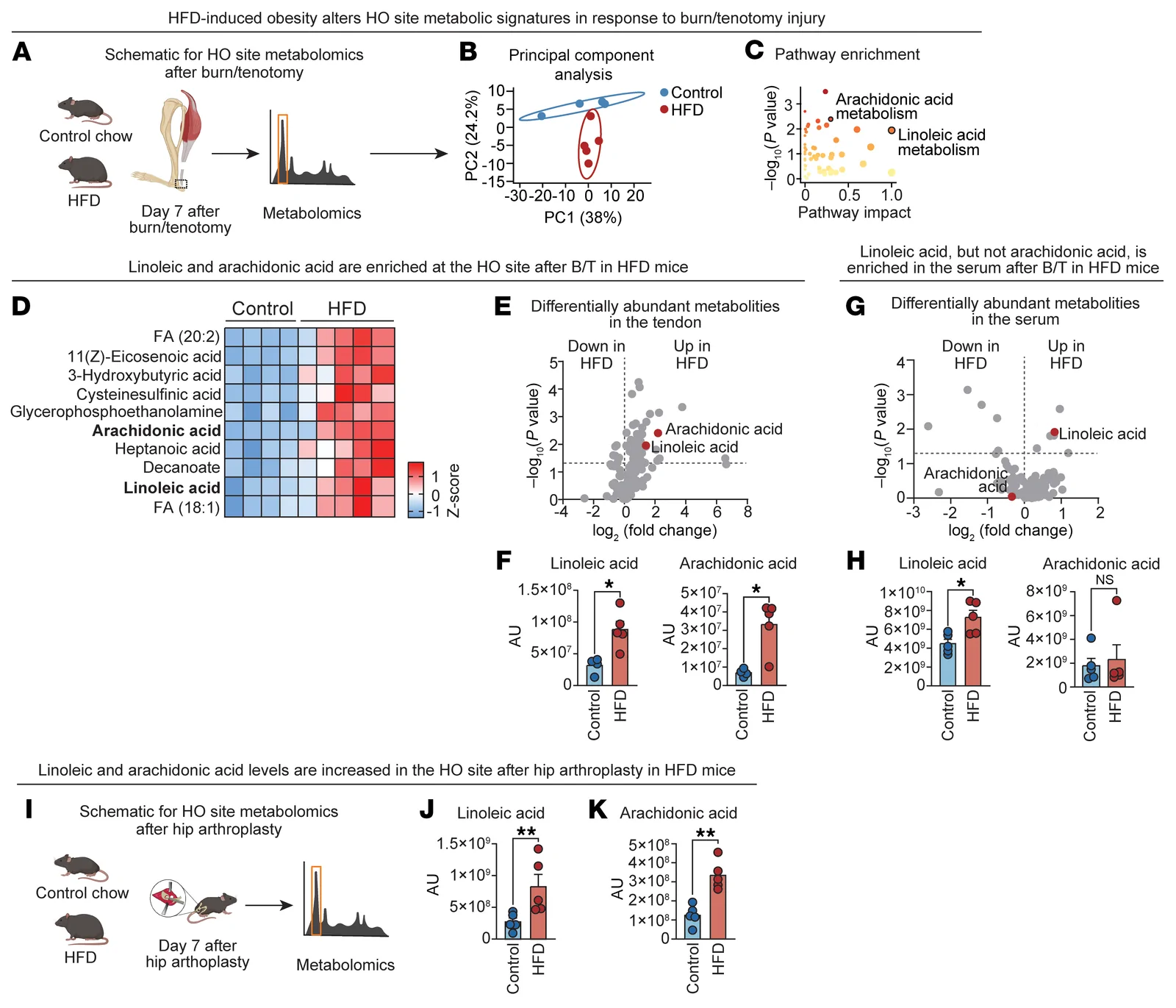
HFD-induced obesity enriches ω-6 fatty acids at the HO site. (**A**) Schematic of metabolomics workflow for the B/T HO site in HFD and control mice (day 7 after injury). (**B**) PCA of HO-site metabolite profiles (PC1, 38%; PC2, 24.2%; *n* = 4–5 mice/group). (**C**) Pathway analysis. (**D**) Heatmap of differential metabolites in HFD versus control (*z* scores; *n* = 4–5 mice/group). (**E**) Volcano plot. (**F**) Quantification of LA and AA abundance at the HO site (*n* = 4–5 mice/group). (**G**) Volcano plot of serum metabolites at day 7 after B/T (LA and AA highlighted; *n* = 5 mice/group). (**H**) Quantification of LA and AA abundance in serum (*n* = 5 mice/group). (**I**) Schematic of metabolomics workflow for hip HO site (day 7 after injury). (**J** and **K**) Quantification of LA and AA abundance at the hip HO site (*n* = 5 mice/group). Means ± SEM are plotted. Statistics: Mann-Whitney test (**F**, **H**, **J**, and **K**). *P < 0.05; **P < 0.01. See also Supplemental Figure 4.

Given the established links between inflammation and lipid metabolism in obesity (34), we next profiled the metabolites in the HO site 7 days after injury (Figure 3A). Untargeted metabolomics revealed distinct clustering of HFD and control samples by principal component analysis (PCA; Figure 3B). Pathway analysis identified LA and AA metabolism among the most significantly upregulated pathways in HFD mice (Figure 3, C–F, and Supplemental Table 2). LA and AA are ω-6 PUFAs, with LA serving as the precursor to AA, a key substrate for inflammatory lipid mediators, including prostaglandins (35). Serum metabolomic analysis showed that HFD increased systemic LA but did not alter AA (Figure 3, G and H). In contrast, only the HFD injury site displayed increased AA (Figure 3F), despite comparable preinjury AA levels across diets (Supplemental Figure 4, A–C). This pattern suggests that HFD elevates LA globally, but AA is selectively enriched at the HO site, suggesting that local conversion of LA to AA primarily occurs at the site of aberrant tissue repair.

These trends were conserved in the HA model, where LA and AA were significantly elevated at the hip HO site (periarticular soft tissue surrounding the injured hip joint) in HFD mice compared with injured controls (Figure 3, I–K). Additionally, we compared the metabolite levels in the injured and contralateral uninjured hips within the same animals. In obese mice, injury increased the abundance of AA at the HO site, without altering LA, while control mice showed no injury-associated changes (Supplemental Figure 4, D and E).

Lipidomics of the HO site 7 days after injury revealed broad diet-dependent lipid remodeling, with HFD HO sites showing increased lysophospholipids across lysophosphatidylethanolamine, lysophosphatidylcholine, and lysophosphatidylinositol subclasses (Supplemental Figure 4, F–J). Several of the most enriched species were lysophospholipids containing AA (20:4) acyl chains (Supplemental Figure 4I). AA-bearing lipids are generated by cytosolic phospholipase A2α–independent (cPLA2α, *Pla2g4a*) cleavage of membrane phospholipids, a process that increases during tissue injury and inflammation (36). Notably, these lipids were elevated only in the HFD HO site, indicating that AA release is specific to obese states. Together, these findings indicate that obesity not only increases ω-6 lipid abundance, but also enhances membrane lipid remodeling, allowing for injury-specific AA release and accumulation within the HO site.

### Myeloid COX-2 promotes local PGE_2_ production and HO in HFD-induced obesity.

**Figure 4:**
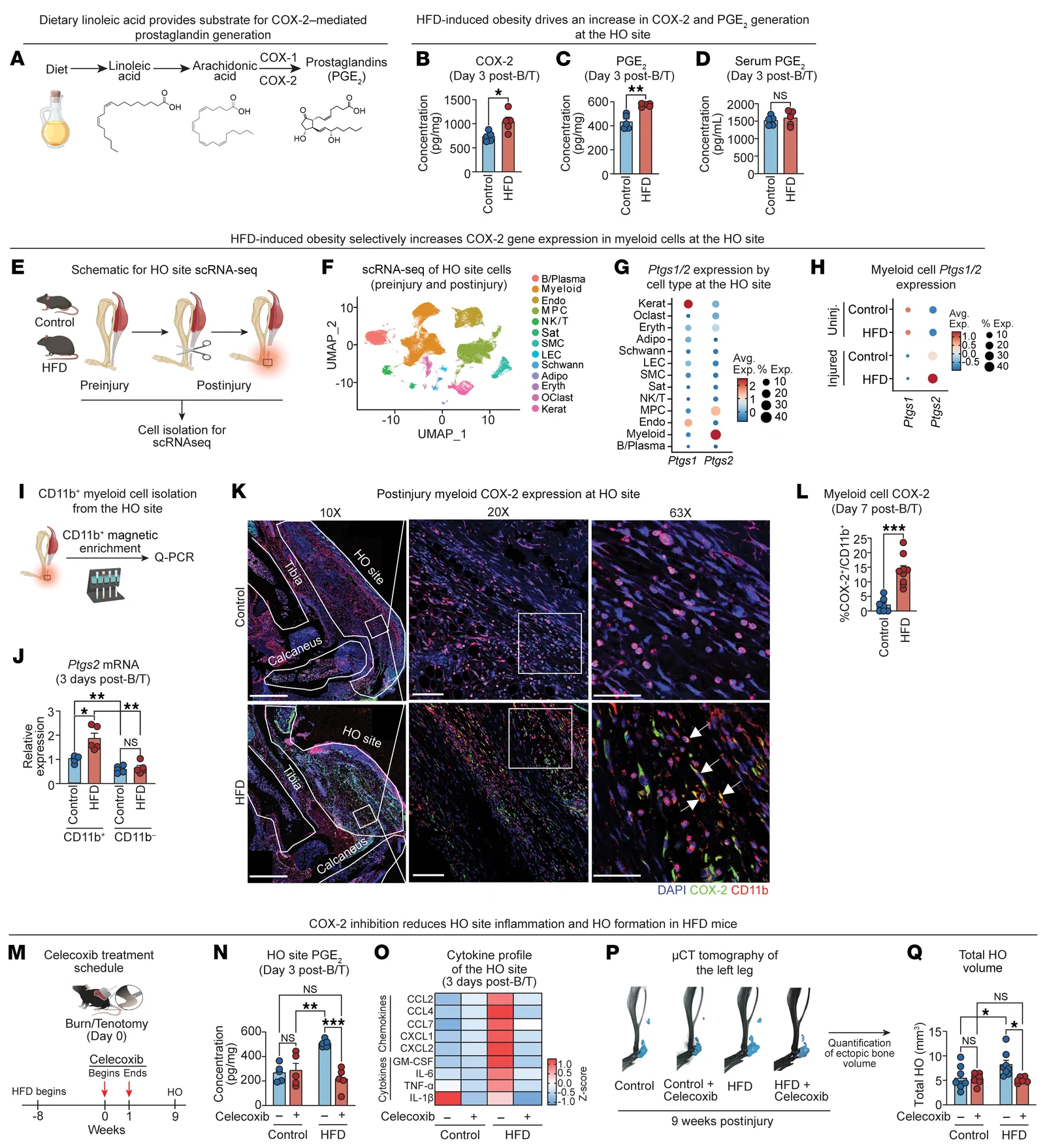
Myeloid COX-2 promotes local PGE_2_ production and HO in obesity. (**A**) Overview: dietary LA from an HFD serves as a precursor for AA, which is metabolized by COX enzymes into prostaglandins, including PGE_2_. (**B**–**D**) COX-2 and PGE_2_ levels at the HO site and serum PGE_2_ levels in HFD and control mice (day 3 after B/T; *n* = 5 mice/group). (**E**) Schematic of scRNA-seq workflow for HO-site tissue before and after B/T injury. (**F**) UMAP of HO-site cell populations annotated by canonical marker expression. (**G** and **H**) *Ptgs1* and *Ptgs2* expression by cell type and within myeloid cells split by diet and injury status. (**I**) Schematic of CD11b^+^ myeloid cell enrichment. (**J**) *Ptgs2* expression in CD11b^+^ myeloid and CD11b^–^ nonmyeloid cells (*n* = 4–5 mice/group). (**K** and **L**) Confocal imaging and quantification of COX-2 colocalization with CD11b^+^ myeloid cells in injured hind limbs at 1 week after B/T (*n* = 3–4 mice/group). Each point represents the proportion of CD11b^+^ cells expressing COX-2 within individual fields of view. White arrows denote Cox2^+^ CD11b^+^ myeloid cells. Scale bars: 1,000 μm (×10), 100 μm (×20), 50 μm (×63). (**M**) Schematic for 1-week celecoxib or vehicle treatment. (**N**) HO-site PGE_2_ levels in control, control + celecoxib, HFD, and HFD + celecoxib mice (day 3 after B/T; *n* = 5 mice/group). (**O**) Heatmap of chemokine and cytokine expression at day 3 after B/T. Control and HFD data from Figure 2B were reanalyzed with celecoxib-treated groups for comparison (*n* = 8–10 mice/group). (**P** and **Q**) μCT images and HO quantification (9 weeks after B/T; *n* = 6–8 mice/group). Statistics: Mann-Whitney test (**B**–**D** and **L**) or 1-way ANOVA (**J**, **N**, and **Q**). Data represent at least 2 independent experiments. Means ± SEM are plotted. *P < 0.05; **P < 0.01; ***P < 0.001. See also Supplemental Figure 5.

Elevated inflammatory signals and increased AA in HFD HO sites suggested enhanced substrate availability for COX activity (Figure 4A) (35). COX enzymes convert AA into prostaglandins, bioactive lipids that regulate inflammation and tissue remodeling (35) with well-established roles in bone formation and fracture repair (37). We next assessed prostaglandin production to determine whether this pathway promotes HO formation in HFD mice. COX-2, the inducible COX isoform activated during injury and inflammatory stress (35), was strongly upregulated at the HFD HO site, accompanied by increased local PGE_2_ levels, while serum PGE_2_ remained unchanged (Figure 4, B–D). These data indicate that HFD-driven enhancement of COX-2 activity was restricted to the HO site.

To more clearly define the cellular and molecular landscape of the HO site in HFD-induced obesity, we performed scRNA-seq of uninjured tendon and HO-site tissue from HFD- and control-fed mice (Figure 4E). Unsupervised clustering and UMAP visualization identified distinct cell populations, with annotations made using canonical marker genes (Figure 4F and Supplemental Figure 5A). Following injury, we found that myeloid cells expressed the highest levels of *Ptgs2* (COX-2) and that *Ptgs2* increased selectively in HFD myeloid cells, whereas *Ptgs1* (COX-1) decreased (Figure 4, G and H). These findings indicate that HFD drives early myeloid-specific activation of COX-2 at the HO site after injury. qPCR of isolated HO-site myeloid cells and immunofluorescence of injured mouse hind limbs supported our findings, demonstrating selective enrichment of COX-2 in CD11b^+^ myeloid cells at the HFD HO site (Figure 4, I–L).

Expression of other key enzymes involved in AA production (delta-5 desaturase *Fads1* and delta-6 desaturase *Fads2*) and prostaglandin synthesis (*Ptges*, microsomal PGE synthase) (38, 39) was increased in HFD myeloid cells at the HO site (Supplemental Figure 5B). *Pla2g4a* (cPLA2α), which releases AA from membrane phospholipids (36), was also significantly elevated (Supplemental Figure 5B), consistent with our lipidomic data showing enrichment of AA-containing lysophospholipids (Supplemental Figure 4I). Importantly, preinjury tendon and serum PGE_2_ levels did not differ between control and HFD mice (Supplemental Figure 5C). These results demonstrate that HFD-induced obesity specifically enhances COX-2 expression in myeloid cells after injury, resulting in increased PGE_2_ production at the HO site.

To test whether COX-2 activity was responsible for the increased HO in HFD mice, we treated HFD mice with celecoxib, an FDA-approved selective COX-2 inhibitor, for 1 week after injury (Figure 4M). We chose this time course to model HO prophylaxis after surgery and minimize off-target effects (40, 41). Celecoxib reduced HO-site and serum PGE_2_ levels (Figure 4N and Supplemental Figure 5D) and selectively lowered local inflammatory cytokines (e.g., CXCL1, IL-6, CCL2, CXCL2, TNF-α) without altering systemic levels (Figure 4O and Supplemental Figure 5E). The antiinflammatory effects of celecoxib were specific to the HFD HO site, consistent with the selective elevation in COX-2 activity observed in HFD mice. Importantly, celecoxib significantly reduced HO in HFD mice but had no effect in control mice (Figure 4, P and Q). These data imply that COX-2 signaling is required for the increased HO in HFD mice and that transient inhibition during the inflammatory phase is sufficient to prevent ectopic bone formation in obesity.

Given the known off-target effects of celecoxib (42), we next tested a cell-specific COX-2 inhibition strategy. Given our scRNA-seq finding that COX-2 is enriched in myeloid cells, we used an FDA-approved polysaccharide nanocarrier composed of dextran, which is selective for these cells (43–45). Local delivery of COX-2 inhibitor-loaded nanoparticles after injury led to reduced HO volume and HO-site PGE_2_ (Supplemental Figure 5, F–H) in HFD mice, suggesting that myeloid targeting of the COX-2/PGE_2_ pathway represents a novel cell-specific therapeutic strategy to attenuate HO.

Consistent with our preclinical findings, TriNetX analysis showed that obese patients who underwent total joint arthroplasty and were prescribed celecoxib postoperatively had reduced HO incidence compared with obese patients not receiving COX-2 inhibition (1.12% vs. 1.46%; *P* = 0.0027; risk ratio = 0.791), with no significant effect in nonobese patients (1.21% vs. 1.46%; *P* = 0.103; risk ratio = 0.828) (Supplemental Figure 5I).

Together, these data show that obesity selectively amplifies myeloid COX-2–mediated conversion of injury-site AA to PGE_2_, promoting inflammation and HO formation. These findings highlight an immunometabolic therapeutic opportunity for obese patients at risk of HO.

### HFD-induced obesity induces an osteochondral transcriptional program in MPCs.

**Figure 5:**
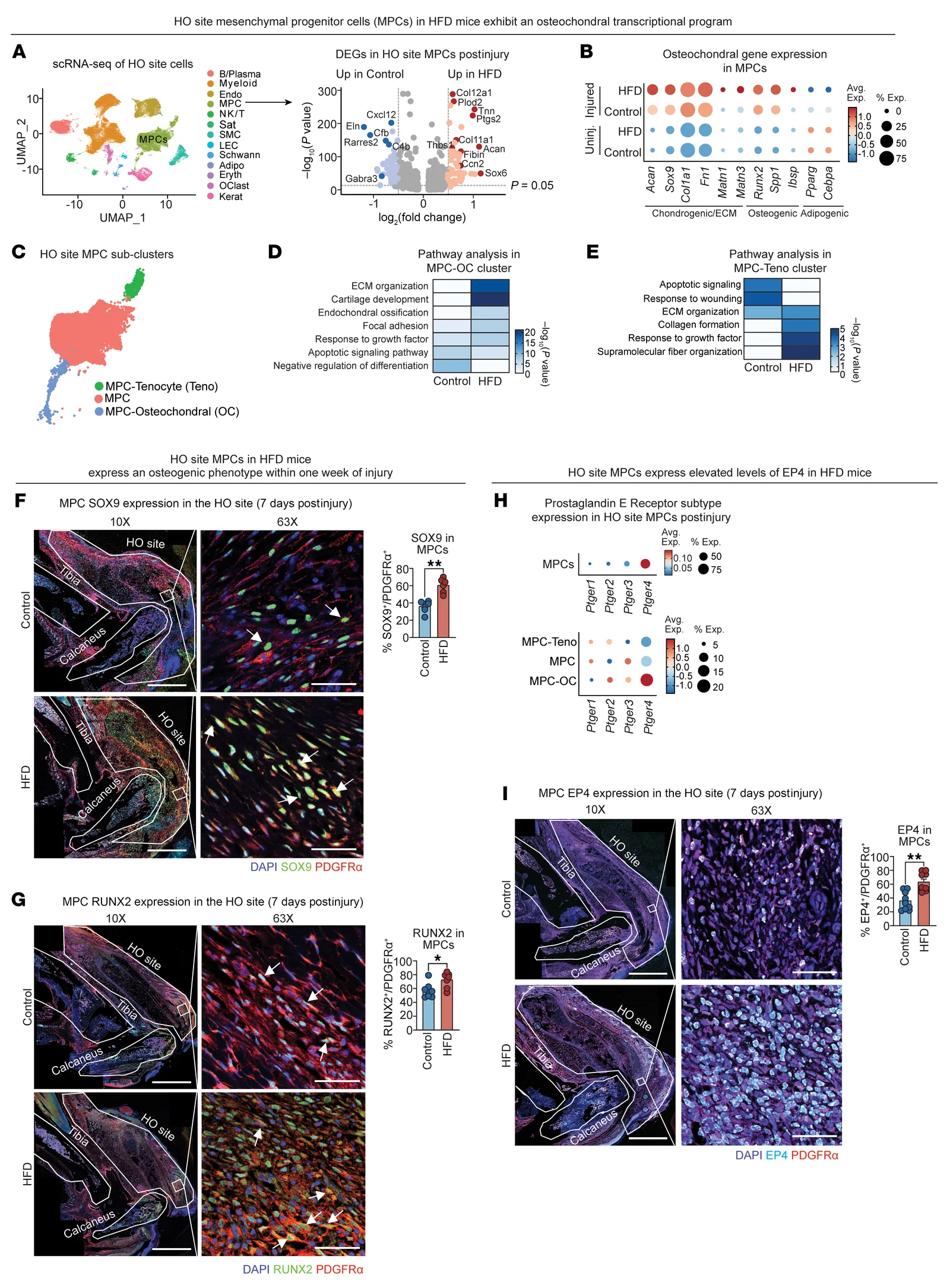
HFD-induced obesity induces an osteochondral transcriptional program in MPCs. (**A**) UMAP of HO-site cell populations (left). Volcano plot (right) showing the top differentially expressed genes in HFD versus control MPCs after injury (red: log_2_FC > 0.5, *P* < 0.001; blue: log_2_FC < –0.5, *P* < 0.001). (**B**) Expression of chondrogenic/ECM, osteogenic, and adipogenic genes in MPCs. (**C**) UMAP of MPC subclusters, including MPC-Tenos and MPC-OCs before and after injury. (**D** and **E**) Pathway analysis in MPC-OCs and MPC-Tenos after injury. (**F**) Confocal images showing colocalization of SOX9 (associated with chondrogenesis) with PDGFRα (MPCs) and quantification in injured HFD and control hind limbs (1 week after B/T; *n* = 3–4 mice/group). Each data point represents the proportion of SOX9^+^PDGFRα^+^ cells within individual fields of view at the HO site. Scale bars: 1,000 μm (×10), 50 μm (×63). White arrows denote SOX9^+^ PDGFRα^+^ MPCs. (**G**) Confocal images showing colocalization of RUNX2 (osteoblast precursors) with PDGFRα and quantification (1 week after B/T; *n* = 3–4 mice/group). Each data point represents the proportion of RUNX2^+^PDGFRα^+^ cells within individual fields of view at the HO site. Scale bars: 1,000 μm (×10), 50 μm (×63). White arrows denote RUNX2^+^ PDGFRα^+^ MPCs. (**H**) PGE_2_ receptor (*Ptger1-4*) gene expression in MPCs and MPC subclusters. (**I**) Confocal images showing colocalization of EP4 (*Ptger4*) with PDGFRα and quantification in injured hind limbs (1 week after B/T; *n* = 3–4 mice/group). Each data point represents the proportion of EP4^+^PDGFRα^+^ cells within individual fields of view at the HO site. Scale bars: 1,000 μm (×10), 50 μm (×63). EP4, PGE_2_ receptor 4. Statistics: Mann-Whitney test (**F**, **G**, and **I**). Data represent at least 2 independent experiments. Means ± SEM are plotted. *P < 0.05; **P < 0.01. See also Supplemental Figure 6.

We next evaluated how the prostaglandin-rich microenvironment at the HFD HO site alters the transcriptomic profile of MPCs, which give rise to ectopic bone (46). scRNA-seq analysis revealed that MPCs from injured HFD HO sites adopted a distinct transcriptional program primed for ectopic bone formation (Figure 5, A and B). These HFD MPCs were enriched for ECM (*Col12a1*, *Plod2*, *Tnn*, *Thbs1*), chondrogenic (*Sox6*, *Acan*), and osteogenic genes (*Runx2*, *Spp1*, *Ibsp*), while adipogenic genes (*Pparg*, *Cebpa*) were suppressed (Figure 5, A and B) (46, 47). In contrast, MPCs from uninjured mice displayed similar gene expression regardless of diet (Figure 5B). GSEA (using normalized enrichment scores) supported these findings, showing that injured HFD MPCs upregulated pathways linked to ectopic bone formation, including ECM remodeling and collagen biosynthesis (Supplemental Figure 6A) (26).

Subclustering of MPCs into MPC-tenocytes (MPC-Tenos), MPC–osteochondral cells (MPC-OCs), and undifferentiated MPCs enabled more detailed analysis of distinct cellular programs after injury, corresponding to tendon repair (MPC-Teno: *Scx*, *Tnmd*) or aberrant osteogenesis (MPC-OC: *Acan*, *Sox6*, *Runx2*) (Figure 5C and Supplemental Figure 6, B–D) (46). HFD MPC-OCs were enriched for ECM organization, cartilage development, endochondral ossification, and growth factor signaling, pathways characteristic of cells primed for osteochondral differentiation (Figure 5D) (26). Even MPC-Teno clusters from HFD mice exhibited increased ECM and collagen-associated pathways (Figure 5E), consistent with a broader shift toward a pro-ossification transcriptional program. Immunofluorescence analysis supported our scRNA-seq findings, revealing an increased proportion of SOX9^+^ (associated with chondrogenesis) and RUNX2^+^ (osteoblast precursor marker) MPCs at the HFD HO site (Figure 5, F and G), consistent with increased osteochondral differentiation. Together, these findings suggest that obesity promotes pro-osteogenic signaling in HO-site MPCs, providing a cellular basis for the increased HO phenotype.

Next, we assessed whether PGE_2_ signaling mediates the transcriptomic changes observed in MPCs. PGE_2_ signals through 4 receptors: EP1, EP2, EP3, and EP4 (*Ptger1-4*) (48). Among *Ptger1-4*, *Ptger4*, implicated by prior studies in bone formation and collagen–ECM interactions (49–51), was the dominant receptor in MPCs and was selectively enriched in MPC-OCs (Figure 5H). We also noted an increased percentage of EP4^+^ MPCs at the HFD HO site (Figure 5I), suggesting enhanced activation of PGE_2_/EP4 signaling.

To determine whether inflammatory COX-2/PGE_2_ activity spatially localizes with osteochondral signaling at the HO site, we performed HD spatial transcriptomics of the injury site from HFD and control mice 7 days after B/T. Spatial analyses revealed higher expression of AA/COX-2 and prostaglandin synthesis pathway genes within the HO-developing site of HFD mice (distal end of transected Achilles tendon) compared with controls (Supplemental Figure 6E). These regions of COX-2^hi^ cells were located near cells enriched for osteochondral differentiation (Supplemental Figure 6E), supporting a distinct immunometabolic microenvironment. Corresponding scRNA-seq analysis showed enrichment of the same gene modules in injured HFD cells (Supplemental Figure 6F). Consistent with our earlier scRNA-seq analyses, demonstrating enhanced *Ptgs2* expression in myeloid cells (Figure 4, H and J) and enrichment of osteochondral genes in HFD MPCs (Figure 5B), these data support a spatially defined COX-2/PGE_2_/EP4 signaling pathway linking myeloid-derived prostaglandins to aberrant MPC differentiation in obesity.

To directly test whether PGE_2_ was sufficient to drive transcriptional changes in MPCs, we treated MPCs isolated from the HO site with PGE_2_ and assessed downstream gene expression (Supplemental Figure 6G). PGE_2_ enhanced the expression of key collagen and ECM-remodeling genes (*Col3a1*, *Itga5*, *Plod2*) and osteochondral genes (*Sox9*, *Runx2*) (Supplemental Figure 6H), similar to the HFD MPC transcriptional program observed in vivo. This effect was significantly reduced by cotreatment with an EP4 inhibitor (L-161,982) (Supplemental Figure 6H) (52). Additionally, *Ptger4* expression was induced with PGE_2_ and suppressed by EP4 inhibition, suggesting that PGE_2_ potentiates the transcription of its receptor on MPCs. These data indicate that PGE_2_/EP4 signaling directly regulates MPC gene expression to promote HO.

### Dietary LA depletion mitigates obesity-associated HO.

Given that LA metabolism and downstream AA/PGE_2_ pathways are increased in HFD-associated HO, we next tested whether dietary LA depletion attenuates HO formation. We generated an isocaloric low-LA HFD (Supplemental Table 1) by replacing soybean oil with a mixture of fats (tallow, palm oil, and olive oil) which are lower in ω-6 PUFAs, while maintaining the same amount of total fat. The amount of dietary LA and the ω-6/ω-3 ratio of the low-LA HFD were similar to the control diet despite the increased total fat content (Supplemental Table 3).

**Figure 6:**
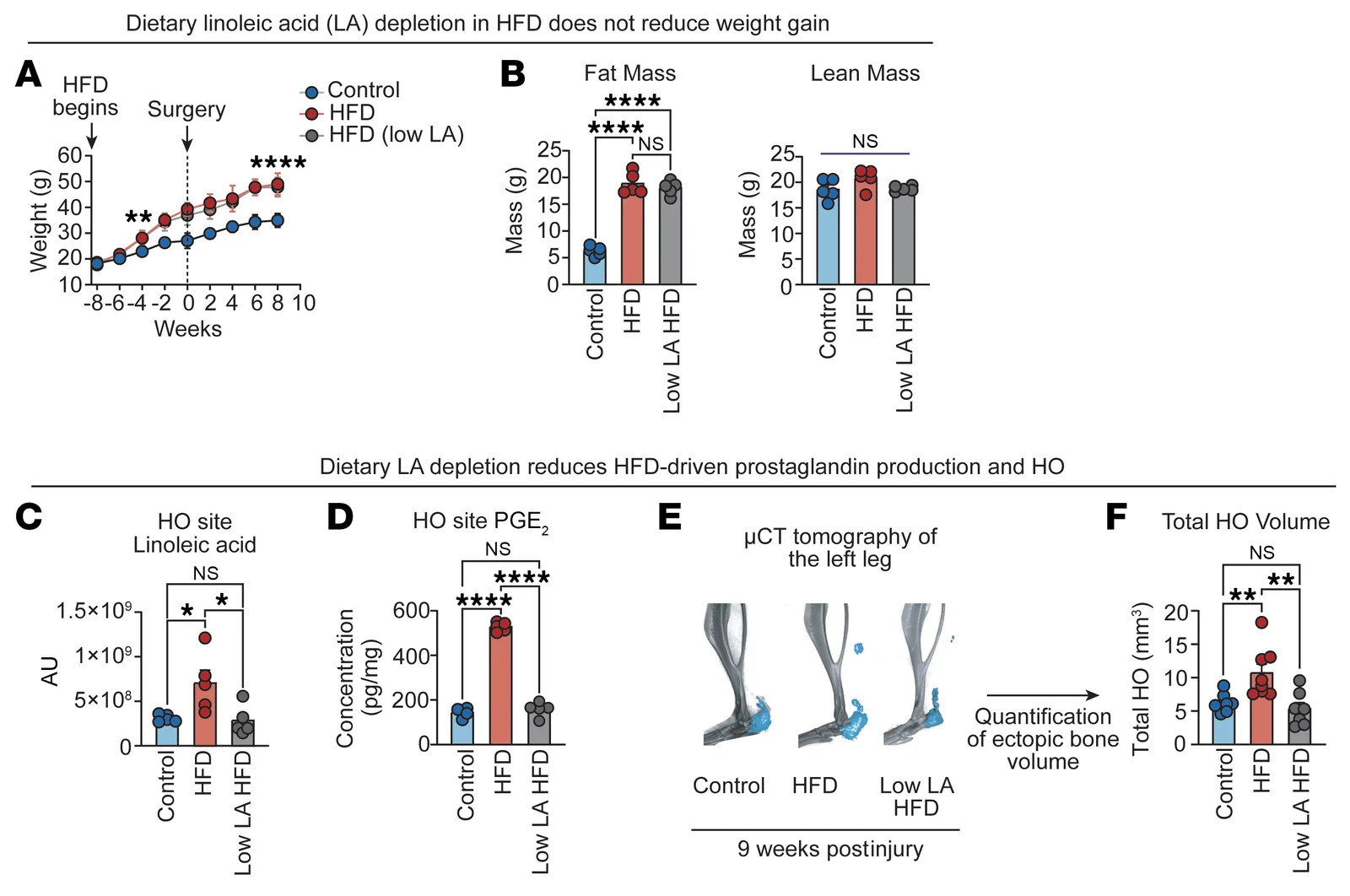
Dietary LA depletion mitigates obesity-associated HO. (**A**) Body weight after diet initiation (*n* = 8 mice/group). Surgery performed at week 0. (**B**) Preinjury fat mass and lean mass (NMR; *n* = 5 mice/group). (**C**) Quantification of LA abundance at the HO site (7 days after B/T; *n* = 4–5 mice/group). (**D**) HO-site PGE_2_ levels normalized to protein (3 days after B/T; *n* = 4–5 mice/group). (**E** and **F**) Representative μCT images and quantification of HO (9 weeks after B/T; *n* = 7–8 mice/group). Data represent at least 2 independent experiments. Means ± SEM are plotted. Statistics: 2-tailed Student’s *t* test (**A**) or 1-way ANOVA (**B**–**D** and **F**). *P < 0.05; **P < 0.01; ****P < 0.0001.

Mice were maintained on the low-LA HFD for 8 weeks before injury and gained body weight and fat mass at a similar distribution and rate to the HFD mice (Figure 6, A and B). Metabolomic analysis of the HO site revealed that LA levels were markedly reduced in low-LA HFD–fed mice and were comparable with controls (Figure 6C). Consistent with reduced ω-6 substrate availability, HO-site PGE_2_ production was also suppressed in low-LA HFD–fed mice (Figure 6D). Accordingly, low-LA HFD–fed mice exhibited significantly reduced total HO volume after injury, comparable with control mice (Figure 6, E and F). Together, these data uncouple diet-induced obesity from ω-6 lipid availability and demonstrate that dietary LA is required to activate COX-2/PGE_2_ signaling and promote aberrant ectopic bone formation.

## Discussion

Obesity is a major risk factor for HO, yet the mechanisms linking metabolic dysfunction to aberrant bone formation remain unclear. In this study, we demonstrated that obesity increased HO by enhancing ω-6 lipid accumulation at the injury site, which altered postinjury tissue repair. Increased LA availability induced myeloid COX-2 activation, increasing PGE_2_ production and activating osteogenic gene expression in MPCs via EP4. Dietary LA depletion or COX-2 inhibition mitigated HO in the setting of an HFD, establishing ω-6 substrate availability as a crucial regulator of ectopic bone formation. Taken together, our data establish an immunometabolic link between dietary lipids and pathologic tissue repair, implicating ω-6 lipids and COX-2/PGE_2_ signaling as therapeutic targets for HO prevention in obesity.

We further showed that HFD-induced obesity leads to spatial and temporal regulation of ω-6 lipids, characterized by elevated systemic and local LA and an injury-dependent increase in AA at the HO site. To our knowledge, this finding was not previously reported in HO or other forms of aberrant tissue repair. These data extend the findings of previous studies implicating ω-6 lipids in inflammatory tissue remodeling (18, 53), demonstrating that injury-site AA enrichment directly impacts pathologic repair. Importantly, AA serves as a substrate for COX-2, which was selectively upregulated in HFD myeloid cells after injury and has well-established roles in immune activation and tissue regeneration (39, 54, 55). A key unresolved question is why myeloid cell recruitment to the injury niche was amplified under HFD. Although our HO-site cytokine analysis revealed increased myeloid chemoattractants, the upstream mechanisms by which the HFD initiates this inflammatory cascade merit further investigation.

Our study also identified PGE_2_ as the central mediator linking ω-6 lipid enrichment to innate immune activation and induction of osteogenic gene expression in MPCs after injury. COX-2–dependent PGE_2_ production is required for the induction of local inflammatory cytokines and chemokines, consistent with previous studies in diabetic wounds and neuronal inflammation (45, 56). We extended these observations by demonstrating that PGE_2_ itself directly induces ECM and osteogenic genes in MPCs, which is attenuated by EP4 inhibition. Across injury models, these MPCs, identified by PDGFRα^+^, represent a conserved population of HO progenitor cells (26, 27), with similar osteochondral features despite anatomical differences. ω-6 Lipid enrichment at both tendon and hip HO sites supports a model in which HFD-driven inflammatory lipid signaling activates PDGFRα^+^ MPCs to drive ectopic bone formation across anatomical sites.

Another key insight is that HFD-induced obesity amplified postinjury HO, but obesity itself was not sufficient to drive this phenotype. Mice fed a low-LA HFD maintained comparable adiposity to the standard HFD mice yet showed significantly reduced HO-site PGE_2_ and ectopic bone volume. These findings indicate that dietary lipid content, rather than excess adiposity alone, determines susceptibility to aberrant repair. This concept aligns with work showing that dietary fat composition shapes immune function (57) and systemic metabolism (19). Despite mixed results from human LA supplementation (58, 59), emerging evidence shows that lowering the ω-6/ω-3 ratio improves postinjury inflammation and recovery (60–62), supporting future investigation of dietary LA depletion as a HO prophylactic strategy.

The translational relevance of this work lies in the identification of ω-6 lipid accumulation as a key metabolic factor rendering the injury site susceptible to COX-2–driven inflammation. Celecoxib reduced HO-site PGE_2_, inflammatory cytokines, and HO in obese mice, paralleling our human retrospective data showing selective efficacy of COX-2 inhibition in obese patients. Prior studies evaluating COX inhibitors for HO prophylaxis have reported variable effectiveness, and there is no consensus on the optimal pharmacologic agent or which patient populations should be treated (10, 11, 63, 64). Our findings suggest that this variability may reflect differences in metabolic state, as increased substrate availability may enhance prostaglandin signaling and HO formation in obesity. These results support stratification by BMI or lipid status to improve therapeutic response and provide a rationale for precision HO prevention, including selective COX-2 inhibition in obese patients and cell-targeted nanoparticle delivery to limit systemic effects.

Several limitations of our study should be acknowledged. Although we observed COX-2 upregulation in CD11b^+^ myeloid cells and identified downstream effects in MPC differentiation, we cannot definitively conclude that myeloid PGE_2_ leads to alterations in MPC gene transcription in vivo. Future studies using conditional genetic models (i.e., myeloid-specific COX-2 deletion or MPC-specific EP4 deletion) should address these remaining questions. Additionally, the upstream triggers of myeloid COX-2 induction after injury in our models remain undefined. Furthermore, while COX-2/PGE_2_ signaling was demonstrated in the B/T model, it was not directly assessed in the HA model and requires further characterization.

In summary, this study defines dietary ω-6 lipid metabolism as a key determinant of HO by linking metabolic state to injury-site inflammation and MPC reprogramming. These findings uncouple obesity from ectopic bone formation and demonstrate that dietary lipid composition, rather than adiposity alone, governs susceptibility to HO. This supports diet-based intervention and selective COX-2 inhibition as rational HO prevention strategies in obese patients. More broadly, this work establishes dietary lipid availability as a modulator of postinjury tissue remodeling, with implications for pathological repair across fibrotic, musculoskeletal, and chronic wound settings.

## Methods

### Sex as a biological variable.

Experiments were performed primarily in male mice because they exhibit a more robust and consistent metabolic response to HFD feeding in our models (65). The pathways examined in the study are not known to be sex-specific and we expect our findings to be relevant to both males and females. However, future studies using female mice will be necessary to determine if the magnitude of the effect differs by sex.

### Animals.

Male C57BL/6J mice (6 weeks old; The Jackson Laboratory, 000664) were maintained on a 12-hour light/12-hour dark cycle with ad libitum access to chow and water. Mice were weighed and randomized before diet assignment. Animals were fed an HFD (D12492), WD (D24090302), control diet (D12450J), or low-LA HFD (D25051601) (Research Diets; Supplemental Table 1) beginning 8 weeks before surgery and continuing throughout the study. Body weight was recorded weekly. Fat and lean mass were measured by NMR (University of Texas Southwestern [UTSW] Metabolic Phenotyping Core) before surgery. Some cohorts of mice received celecoxib (MedChemExpress, HY-14398) (30 mg/kg, i.p.) or vehicle for 7 days after injury. Additional cohorts received dextran-conjugated lumiracoxib polysaccharide nanoparticles (16 mg/kg, s.c.) or dextran (Sigma-Aldrich, 31390) daily for 1 week after B/T (45).

### Surgical procedures.

For B/T, a partial thickness dorsal burn and Achilles tenotomy were performed according to previous studies (25, 26). Following anesthesia, a heated aluminum block (60°C) was applied dorsally for 18 seconds to achieve a partial thickness burn (over 30% of the total body surface area). The Achilles tendon was transected, and the incision was closed with 5-0 Vicryl. 1.2 mg/kg buprenorphine SR was given postoperatively. For subsequent analyses, tissues were harvested from the injured Achilles tendon and the surrounding soft tissue where ectopic bone forms, referred to as the injury or HO site.

For HA, acetabular reaming was performed as described previously (27). After anesthesia, the left hip was exposed laterally. Next, a capsulotomy and femoral head dislocation were performed to allow acetabular reaming with a micropower drill. The incisions were washed and closed with 4-0 Vicryl. Tissues were harvested from the periarticular soft tissue surrounding the injured joint where ectopic bone forms, referred to as the hip HO site.

### μCT.

Hind limb and hip samples were harvested at 9 weeks after B/T or 12 weeks after HA. Samples were fixed in 4% paraformaldehyde for 24 hours and washed in PBS. μCT imaging was performed using a nanoScan PET/CT system (Mediso), and HO volume was quantified in Dragonfly (Object Research Systems) by a blinded operator (66).

### Histology and immunofluorescence staining.

Samples were fixed in 4% paraformaldehyde for 24 hours at 4°C and decalcified in 14% EDTA for 5 weeks. Tissues were paraffin embedded, sectioned, deparaffinized, and rehydrated. After antigen retrieval in Tris-EDTA buffer (pH 9.0) and blocking in 10% donkey serum for 1 hour, sections were incubated overnight at 4°C with primary antibodies: rabbit anti–COX-2 (Cell Signaling Technology, 12282S), rabbit anti-Sox9 (Abcam, ab185966), rabbit anti-Runx2 (Abcam, ab192256), rabbit anti-Ptger4 (BioLegend, 100222), goat anti-PDGFRα (R&D Systems, AF1062), and anti-rat CD11b (Abcam, ab8878). This was followed by staining with secondary antibodies: donkey anti-rat IgG, Alexa Fluor 647 (Thermo Fisher Scientific, A48272TR), donkey anti-rabbit IgG, Alexa Fluor 488 (Thermo Fisher Scientific, A-21206), donkey anti-rabbit IgG, Alexa Fluor 555 (Thermo Fisher Scientific, A-31572), and donkey anti-goat IgG, Alexa Fluor 488 (Thermo Fisher Scientific, A-11055) for 1 hour and DAPI Fluoromount-G (Southern Biotechnology) for nuclear staining. Images were acquired on a Leica SP8 confocal microscope and quantified in ImageJ (NIH). Safranin O, Masson’s trichrome, and H&E slides were scanned with a Hamamatsu NanoZoomer 2.0-HT.

### Range of motion.

For ankle and hip range of motion, a constant force was applied to induce extension or abduction, respectively, followed by x-ray imaging. Ankle range of motion was measured as described previously (26). Hip range of motion was quantified as the angle between the femoral shaft and ipsilateral pelvis.

### Burn wound healing analysis.

Sequential photographs were taken to quantify wound area. A ruler was used for calibration. Wound area was traced and measured in ImageJ and normalized to initial size.

### Functional behavior assays.

Behavior tests were performed at the UTSW Rodent Behavior Core. Locomotor activity was recorded for 60 minutes using a photobeam system. Rotarod testing was performed at 4–40 rpm acceleration over 5 minutes (4 times/day for 2 days). Digigait was assessed at 20 cm/s to record gait measurements and calculate hind limb loading scores (percent stance per stride multiplied by paw area).

### Black box analysis.

Mice were recorded for 6 minutes in a dark black box (Black Box Bio) under controlled lighting. Recordings were analyzed with Palmreader software to assess hind limb pressure index (30).

### TriNetX.

We identified adult patients (ages 18 to 75 years) undergoing total hip or knee arthroplasty in the TriNetX database. HO was defined using ICD codes for HO, hypertrophy of bone, or calcification and ossification of muscle occurring 1 day to 5 years after surgery. Obesity was defined as BMI greater than 35 kg/m^2^ and normal BMI as 18.5–24.9 kg/m^2^. HO incidence was compared between obese and nonobese patients, and the association of celecoxib use within 1 month after surgery and HO risk was evaluated in each group.

### Luminex.

Cytokines in HO-site lysates and serum were measured by the UTSW Microarray and Immune Phenotyping Core. Tissue was snap-frozen and homogenized in lysis buffer (Cell Lysis Buffer 2, R&D Systems, 895347) with protease and phosphatase inhibitors. Diluted samples were analyzed on a Bio-Plex 200 platform, and concentrations were normalized to total protein.

### Flow cytometry.

HO-site tissue was digested into single-cell suspensions as previously described (32). Suspensions were incubated with live/dead viability dye (Thermo Fisher Scientific, 50-205-0581) and anti-CD16/32 (Fc Block) for 10 minutes at 4°C. Staining was performed with fluorophore-conjugated antibodies (anti-mouse CD45, BioLegend, 103151; anti-mouse F4/80, BioLegend, 123116; anti-mouse Ly-6C, BD Biosciences, 560596; anti-mouse Ly-6G, BioLegend, 127643; anti-mouse/human CD11b; BioLegend; 101216) for 25 minutes at 4°C. After washing, samples were acquired on a NovoCyte 3005 flow cytometer and analyzed using NovoExpress software.

### Metabolomic profiling.

HO-site tissue and serum were flash-frozen and processed using published protocols (67, 68). Metabolites were extracted in 80% methanol, normalized, and dried down before reconstitution in 80% acetonitrile. Liquid chromatography–mass spectrometry was performed at the CRI Metabolomics Core (UTSW) using an Exploris 480 mass spectrometer (Thermo Fisher Scientific). Untargeted pathway analyses were performed in MetaboAnalyst 5.0 (https://www.metaboanalyst.ca/). Data were normalized by sum and autoscaled.

### Lipidomic profiling.

Serum and tissue lipids were extracted using a modified Folch protocol and analyzed on a Lumos 1M mass spectrometer (69). The lipidomics method records precursor and product ion spectra of lipids at the MS2 and MS3 level. Compound Discoverer 3.4 (Thermo Fisher Scientific) using the LipidSearch (Thermo Fisher Scientific) algorithm automatically assigned lipid IDs based on the spectra collected. Lipid assignments were reviewed manually for product ion spectra consistent with those from purified standards.

### COX-2 and PGE_2_ assay.

COX-2 was measured with a COX-2 ELISA (Abcam, ab210574) and PGE_2_ with a PGE_2_ ELISA (Cayman Chemical, 500141) according to the manufacturers’ protocols. Values were normalized to total protein.

### scRNA-seq.

scRNA-seq was performed on cells isolated from the injury site at 0, 3, and 7 days (*n* = 4 biological replicates pooled per group) after B/T on HFD-fed and age-matched controls, following previous protocols (32). Briefly, approximately 1 billion reads were generated per group using the NovaSeq X platform and the Chromium GEM Single Cell 3′ Kit v3.1 (PN-1000121). The data were preprocessed using Cell Ranger (10x Genomics) to align reads to the mm10-2020-A genome, achieving a 94% alignment rate and a median of 2,200 genes detected per cell. Quality control and downstream analyses were performed using Seurat v5 (https://satijalab.org/seurat/) (70). Cells with feature counts below the 0.04 quantile or above the 0.96 quantile, RNA counts greater than 60,000, or mitochondrial read content exceeding 15% were filtered out. Subsequent analyses included cell-cycle scoring, normalization using SCTransform with regression of cell-cycle score differences, integration using the Harmony method, dimensionality reduction (PCA and UMAP), unsupervised clustering (resolution = 0.5), and differential expression analysis. Differentially expressed genes between aligned clusters were identified using a negative binomial test.

### Spatial transcriptomics.

Injury-site tissue from HFD and control mice (*n* = 2 per group) were excised 7 days after B/T, fixed in 4% PFA, and paraffin embedded. Visium HD data were generated at the UTSW Microbiome Core according to 10x Genomics (CG000685). Reads were demultiplexed and aligned to the mouse (mm10-2020-A) genome using SpaceRanger v3.1.2. Data (8 μm bins) were analyzed in Seurat. Spots with zero detected counts and genes expressed in fewer than 0.3% of spatial spots were excluded. Expression values were normalized using log normalization, followed by variable feature selection, data scaling, and PCA. To enable scalable analysis of high-resolution spatial datasets, a representative subset of 50,000 spatial spots was selected using leverage score–based sketching. Clustering and UMAP embeddings were computed on the sketched dataset and subsequently projected to the full dataset. Cell-type deconvolution of spatial spots was performed using Seurat’s FindTransferAnchors with annotated scRNA-seq, and inferred cell-type labels were projected to all spatial spots. Differential gene expression analyses and spatial expression plots (SpatialFeaturePlot) were performed.

### Myeloid cell enrichment.

Immune cells from the HO site underwent magnetic enrichment for CD11b^+^ myeloid cell isolation using Miltenyi Microbeads (Miltenyi Biotec,130-126-725) and LS columns following the manufacturer’s protocol.

### qPCR.

RNA was isolated and extracted (RNeasy Plus Universal Mini Kit) and converted to cDNA using M-MLV Reverse Transcriptase (Thermo Fisher Scientific). qPCR with TaqMan probes (Thermo Fischer Scientific, 18S, Mm03928990_g1; Ptgs2, Mm00478374_m1; Fads1, Mm00507605_m1; Fads2, Mm00517221_m1; Pla2g4a, Mm00447040_m1; Ptges, Mm00452105_m1; Col3a1, Mm00802300_m1; Itga5, Mm00439797_m1; Plod2, Mm00478767_m1; Sox9, Mm00448840_m1; Runx2, Mm00501584_m1) and TaqMan Universal PCR Master Mix (Thermo Fisher Scientific) was performed on a QuantStudio 7 Flex Real-Time PCR System. Relative transcript levels were analyzed using the ΔΔCt method and were normalized to 18S rRNA levels.

### MPC isolation and treatment with PGE_2_.

HO-site tissue was digested as described previously (26). Adherent cells (MPCs) were cultured in DMEM with 20% FBS. Cells were treated with PGE_2_ (1 μM), PGE_2_ (1 μM), and an EP4 receptor antagonist (L-161,982; 10 μM) (52) or vehicle for 24 hours and collected for RT-qPCR.

### Statistics.

Quantitative data are expressed as the mean ± SEM. *P* < 0.05, *P* < 0.01, *P* < 0.001, and *P* < 0.001 were considered significant. The number of replicates is indicated in the figure legends and reflects biologically independent experiments conducted at different times. Data were analyzed with GraphPad Prism 10.5.0 using a 2-tailed Mann-Whitney test or 1-way ANOVA with Tukey’s multiple-comparison test. Additional statistical tests, if applied, are specified in the figure legends.

### Study approval.

All animal studies were approved by the IACUC at the University of Texas Southwestern Medical Center (APN 2021-103130) and conducted in accordance with the Guide for the Care and Use of Laboratory Animals.

### Data availability.

scRNA-seq and spatial transcriptomics data are available from the Gene Expression Omnibus repository under accession number GSE317906. All data supporting the results of the study are provided in the main text and supplemental materials. Numerical values for all plotted data are included in the Supporting Data Values file.

## Author contributions

SLM and BL conceptualized the study. SLM, TS, CAP, AD, OG, DF, LGZ, and TPM performed the research. SLM, MM, TS, SK, LGZ, and TPM analyzed the data. SLM and BL wrote the original draft of the paper. ZL, AWJ, GH, AMS, KAG, TPM, and RJT provided reagents and gave input on the experiments for this study. SLM and BL reviewed and edited the manuscript.

## Conflict of interest

The authors have declared that no conflict of interest exists.

## Funding support

This work is the result of NIH funding, in whole or in part, and is subject to the NIH Public Access Policy. Through acceptance of this federal funding, the NIH has been given a right to make the work publicly available in PubMed Central.

- NIH National Institute of Arthritis and Musculoskeletal and Skin Diseases (NIAMS) R01AR079863 (KAG and BL).
- NIH NIAMS F31 1F31AR086578-01 (SLM).
- Cancer Prevention and Research Institute of Texas Core Facilities Support Award RP240494 (LGZ and TPM).
- US Dept. of Defense grant HT94252310535 (RJT).

## Supplementary Material

Supplemental data

Supplemental video 1

Supplemental video 2

Supplemental video 3

Supplemental video 4

Supporting data values

## Version 1 06/11/2026

In-Press Preview

## Version 2 09/01/2026

Electronic publication
