# Supplementary material for: Dietary omega-6 lipids promote postinjury aberrant bone formation in obesity
Source: J Clin Invest. 2026 Jun 11;136(17):e205829. doi: 10.1172/JCI205829 (PMC13528920; doi:10.1172/JCI205829)
Supplement: Supplementary file 1 [file jci-136-205829-s079.pdf]

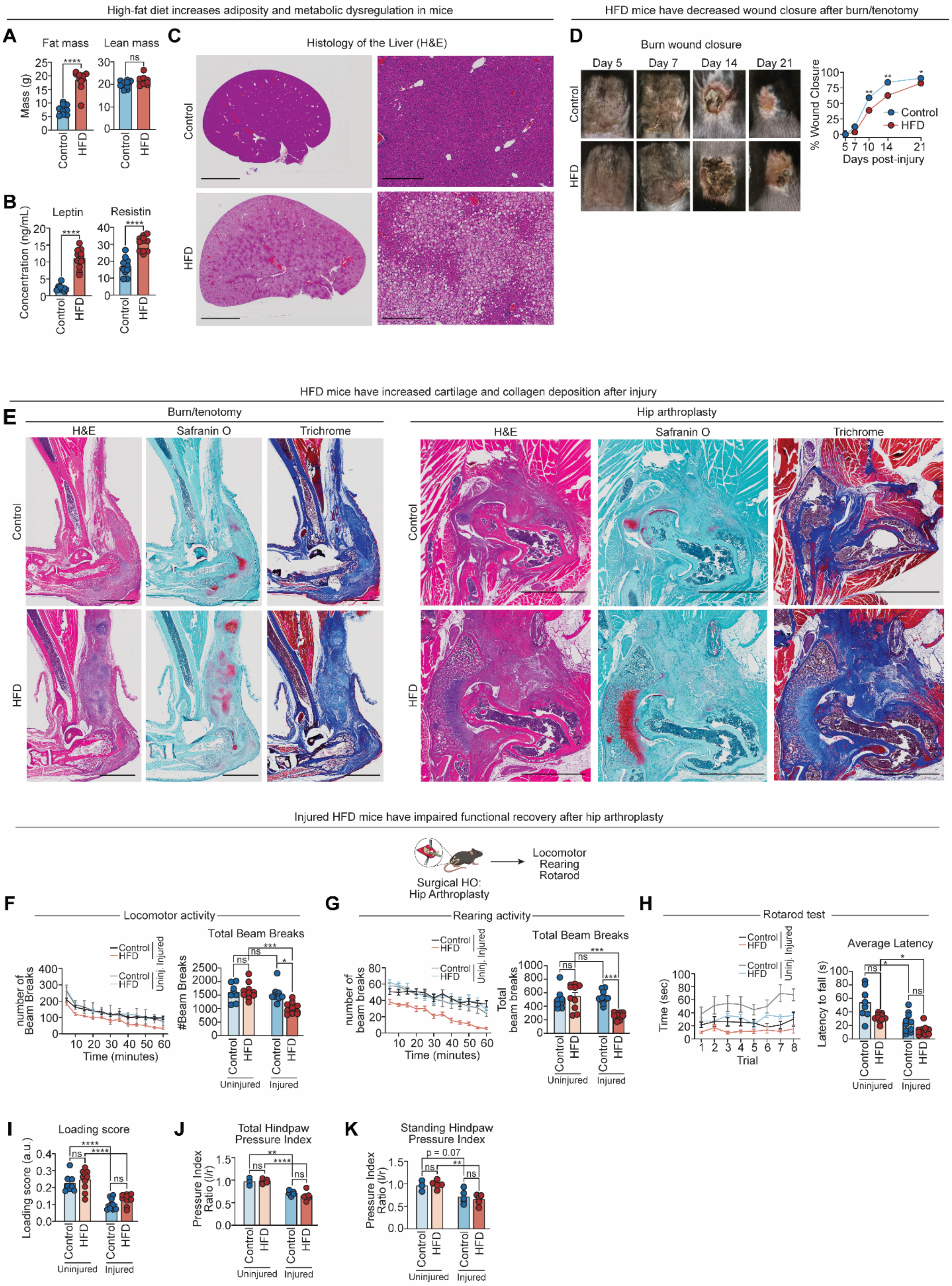

High-fat diet increases adiposity and metabolic dysregulation in mice
HFD mice have decreased wound closure after burn/tenotomy
A
Fat mass
Lean mass
Mass (g)
Control
HFD
****
ns
B
Leptin
Resistin
Concentration (ng/mL)
C
Histology of the Liver (H&E)
Control
HFD
D
Burn wound closure
Day 5
Day 7
Day 14
Day 21
% Wound Closure
Days post-injury
Control
HFD
HFD mice have increased cartilage and collagen deposition after injury
E
Burn/tenotomy
Hip arthroplasty
H&E
Safranin O
Trichrome
Injured HFD mice have impaired functional recovery after hip arthroplasty
Surgical HO:
Hip Arthroplasty
Locomotor
Rearing
Rotarod
F
Locomotor activity
Total Beam Breaks
number of Beam Breaks
#Beam Breaks
Time (minutes)
Uninjured
Injured
G
Rearing activity
Total Beam Breaks
number of beam breaks
Total beam breaks
Time (minutes)
H
Rotarod test
Average Latency
Time (sec)
Trial
Latency to fall (s)
I
Loading score
Loading score (a.u.)
J
Total Hindpaw Pressure Index
Pressure Index Ratio (l/r)
K
Standing Hindpaw Pressure Index
p = 0.07

**Supplemental Figure 1: Metabolic and functional characterization of control and HFD mice pre- and post-injury.**

**(A)** Fat mass and lean mass pre-injury measured with NMR in HFD and control mice (n = 10 mice/group).
**(B)** Serum leptin and resistin levels pre-injury (n = 10-11 mice/group).
**(C)** Representative liver H&E sections at 9 weeks post-B/T. Low- and high-magnification images are shown. Scale bars = 5mm (left), 500 μM (right).
**(D)** Time course of percentage of burn wound closure normalized to baseline wound area (n = 3 mice/group).
**(E)** H&E, Safranin O, and Trichrome staining in the B/T (left panel) and HA (right panel) model at 3 weeks post-injury in HFD and control mice. Images demonstrate cartilage and collagen deposition in the injured limb or hip. Scale bars = 2.5 mm.
**(F)** Locomotor activities were measured as number of beam breaks in control and HFD mice with and without HA at 6 weeks post-HA. Left: time course of activity for the 4 groups. Right: total mean activity per mouse (n = 8-10 mice/group).
**(G)** Rearing activities were measured as number of beam breaks at 6 weeks post-HA. Left: time course of rearing for the 4 groups. Right: total mean rearing per mouse (n = 8-10 mice/group).
**(H)** Rotarod testing showing the latency to fall of the rod (sec) at 6 weeks post-HA. Left: time course of rotarod performance across 8 trials. Right: mean latency to fall per mouse (n = 8-10 mice/group).
**(I)** Hindlimb loading score as a proxy for mechanical load (n = 8-10 mice/group).
**(J)** Total hindpaw pressure index (left/right hindpaw) (n = 3-5 mice/group).
**(K)** Standing hindpaw pressure index (left/right hindpaw) (n = 3-5 mice/group).
HO, heterotopic ossification; B/T, burn/tenotomy; HA, Hip Arthroplasty; HFD, high-fat diet; NMR; nuclear magnetic resonance; μCT, micro-computed tomography; Hematoxylin and eosin, H&E. Means ± SEM are plotted. All experiments are representative of at least two independent experiments. Statistics: Mann-Whitney (A, B), two-tailed Student's t test (D), Kruskal-Wallis (F, H), or one-way ANOVA (G, I, J, K). *$p < 0.05$; **$p < 0.01$; ***$p < 0.001$; ****$p < 0.0001$; ns, not significant.

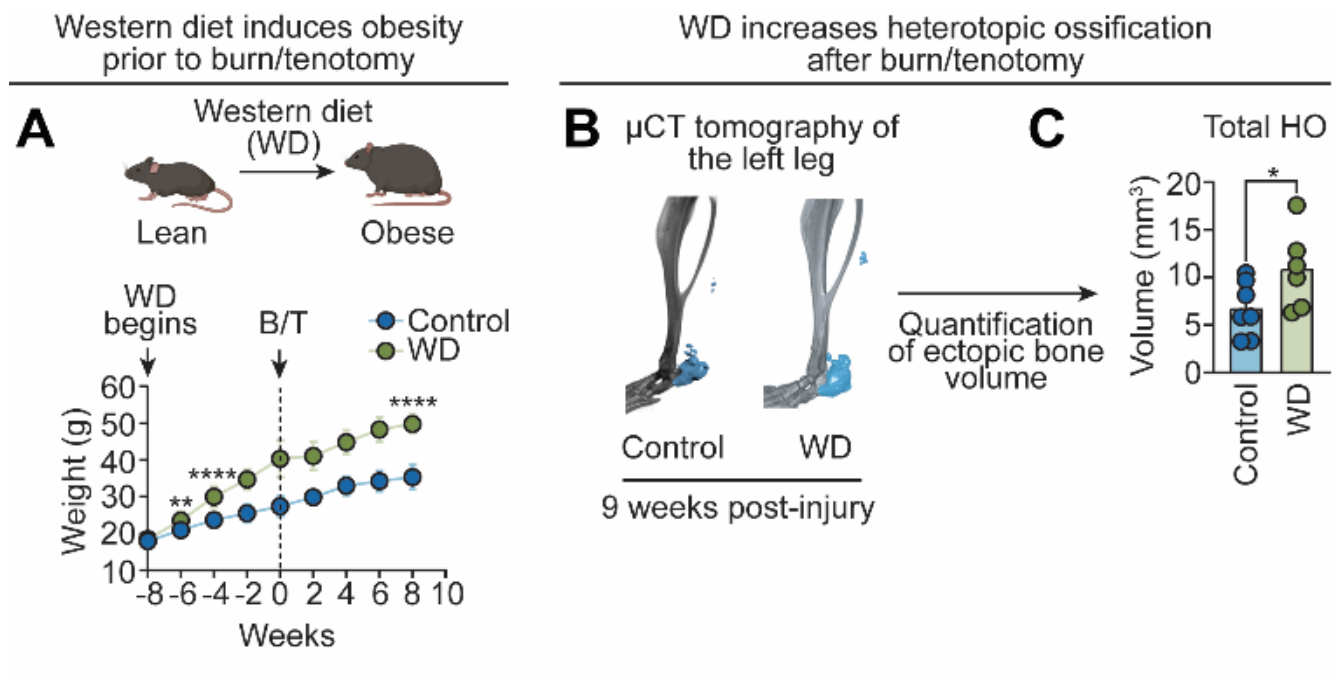


**Supplemental Figure 2: Western diet promotes HO.**
**(A)** Weights of Western diet (WD) and control mice after diet initiation. Mice were started on the WD 8 weeks prior to undergoing B/T and were followed for 9 weeks (n = 8-10 mice/group).
**(B)** Representative μCT images of ankle HO in the injured leg of WD and control mice 9 weeks after B/T.
**(C)** μCT quantification of total HO volume at 9 weeks post-injury (n = 6-7 mice/group).
HO, heterotopic ossification; WD, Western Diet; B/T, burn/tenotomy; μCT, micro-computed tomography. Means ± SEM are plotted. All experiments are representative of at least two independent experiments. Statistics: two-tailed Student's t test (A) or Mann-Whitney (C). *$p < 0.05$; **$p < 0.01$; ****$p < 0.0001$.

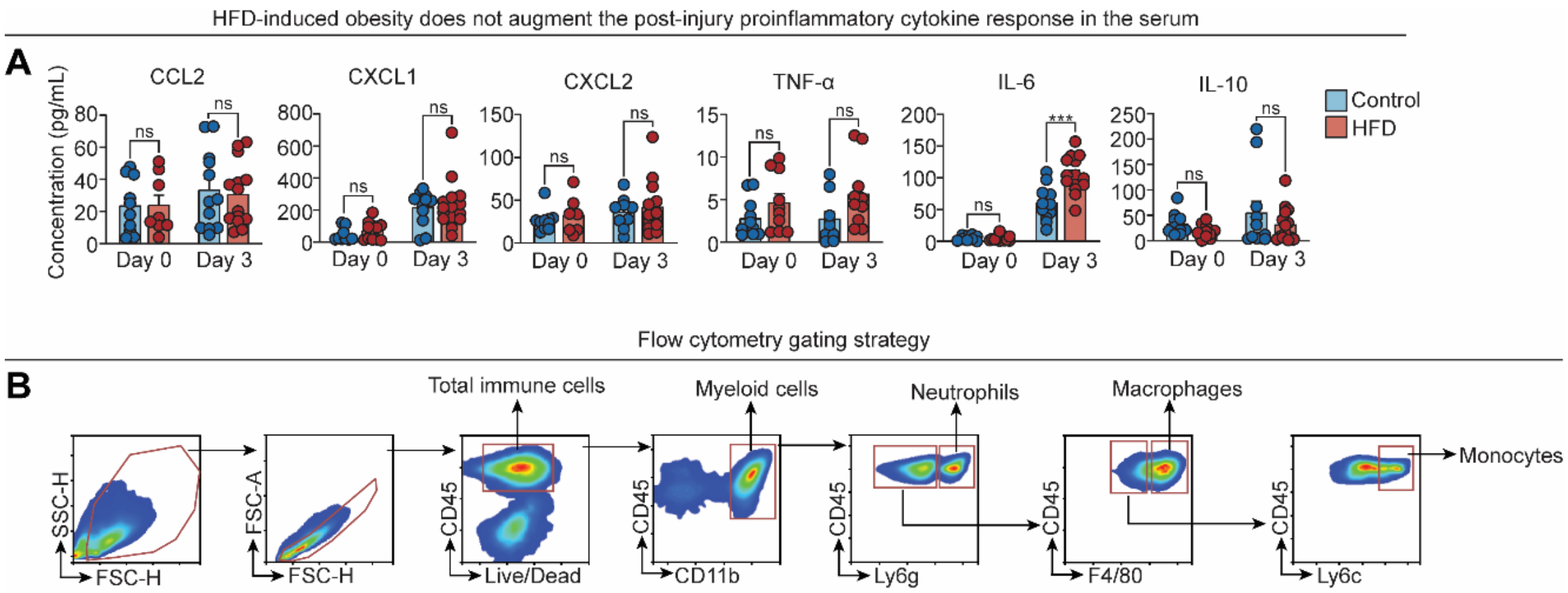


**Supplemental Figure 3: Serum cytokine profiling and flow cytometry gating strategy for HO-site immune cells.**

**(A)** Serum CCL2, CXCL1, CXCL2, TNFα, IL-6, and IL-10 (day 0 and day 3 post-B/T) in HFD and control mice. Cytokine levels were compared between dietary conditions at each time point (day 0 or day 3 post-B/T) (n = 9-10 mice/group).

**(B)** Gating strategy for identifying immune populations in the injury site. Cell subsets include total immune cells (live $CD45^{+}$), neutrophils (live $CD45^{+}$ $CD11b^{+}Ly6g^{+}$), macrophages (live $CD45^{+}CD11b^{+}$ $Ly6g^{-}$ $F4/80^{+}$), and monocytes (live $CD45^{+}CD11b^{+}Ly6g^{-}F4/80^{-}Ly6c^{+}$).

HFD, high-fat diet; B/T, burn/tenotomy. Means ± SEM are plotted. All experiments are representative of at least two independent experiments. Statistics: Mann-Whitney test (A). ***$p < 0.001$; ns, not significant.

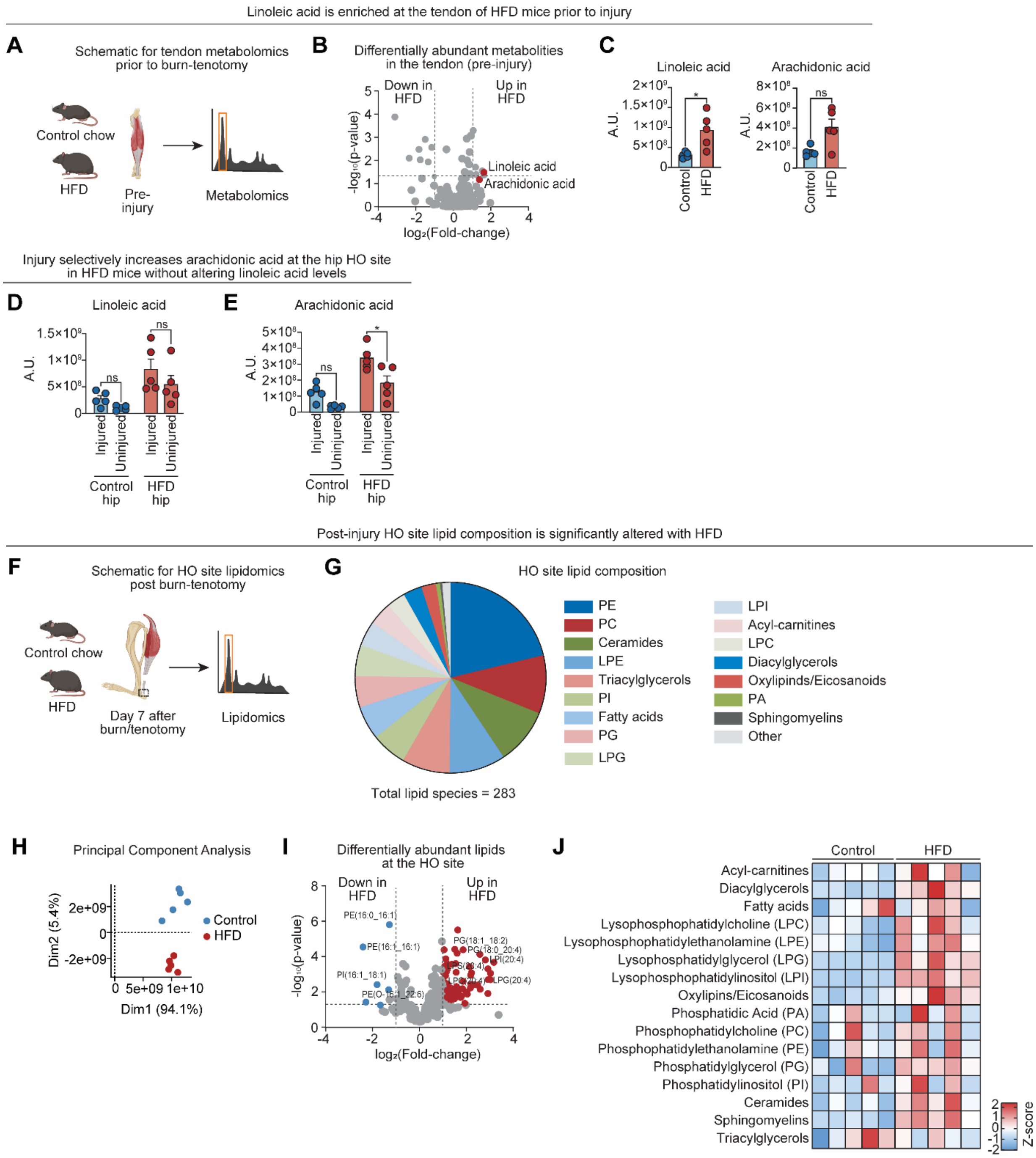


**Supplemental Figure 4: Baseline tendon metabolomics and post-injury lipid alterations at the HO site.**

**(A)** Experimental schematic of metabolomic characterization of uninjured tendons in HFD and control mice at day 0 before B/T.

**(B)** Volcano plot of uninjured tendon metabolites with LA and AA metabolites noted in red. The horizontal line indicates statistical significance ($p < 0.05$). The vertical lines denote $log_2FC = -1$ and +1.

**(C)** Quantification of LA and AA abundance before B/T (n = 5 mice/group).

**(D)** Quantification of LA abundance in the injured and uninjured hips at day 7 post-HA; Metabolite levels were compared between injured and uninjured hips within each dietary condition (n = 5 mice/group). Data from Figure 3J were replotted to allow direct comparison with uninjured groups.
**(E)** Quantification of AA abundance in the injured and uninjured hips at day 7 post-HA; Metabolite levels were compared between injured and uninjured hips within each dietary condition (n = 5 mice/group). Data from Figure 3K were replotted to allow direct comparison with uninjured groups.
**(F)** Experimental schematic of lipidomic characterization of control and HFD HO sites at day 7 post-B/T.
**(G)** Pie chart showing the distribution of HO-site lipid classes in the dataset. Total lipid species = 283.
**(H)** PCA showing distinct lipid profiles in the HO site. PC1 and PC2 explain 94.1% and 5.4% of total variance respectively (n = 5 mice/group).
**(I)** Volcano plot of HFD vs. control HO-site lipids. The horizontal line indicates statistical significance ($p < 0.05$). The vertical lines denote $\log_2FC = -1$ and $+1$. Red dots (up in HFD) signify lipids which have $p < 0.05$ and $\log_2FC > 1$ and blue dots (down in HFD) signify lipids which have $p < 0.05$ and $\log_2FC < -1$. Top lipids in HFD contain AA-acyl chains: LPG 20:4, LPS 20:4, LPI 20:4, LPG 20:4.
**(J)** Heatmap of HO-site lipid classes. Values are normalized as z-scores (n = 5 mice/group).
B/T, burn/tenotomy; HFD, high-fat diet; LA, linoleic acid; AA, arachidonic acid; HA, hip arthroplasty; PCA; Principal component analysis; FC, Fold-change. Means ± SEM are plotted. All experiments are representative of at least two independent experiments. Statistics: Mann-Whitney test (C) and one-way ANOVA (D, E). *$p < 0.05$; ns, not significant.

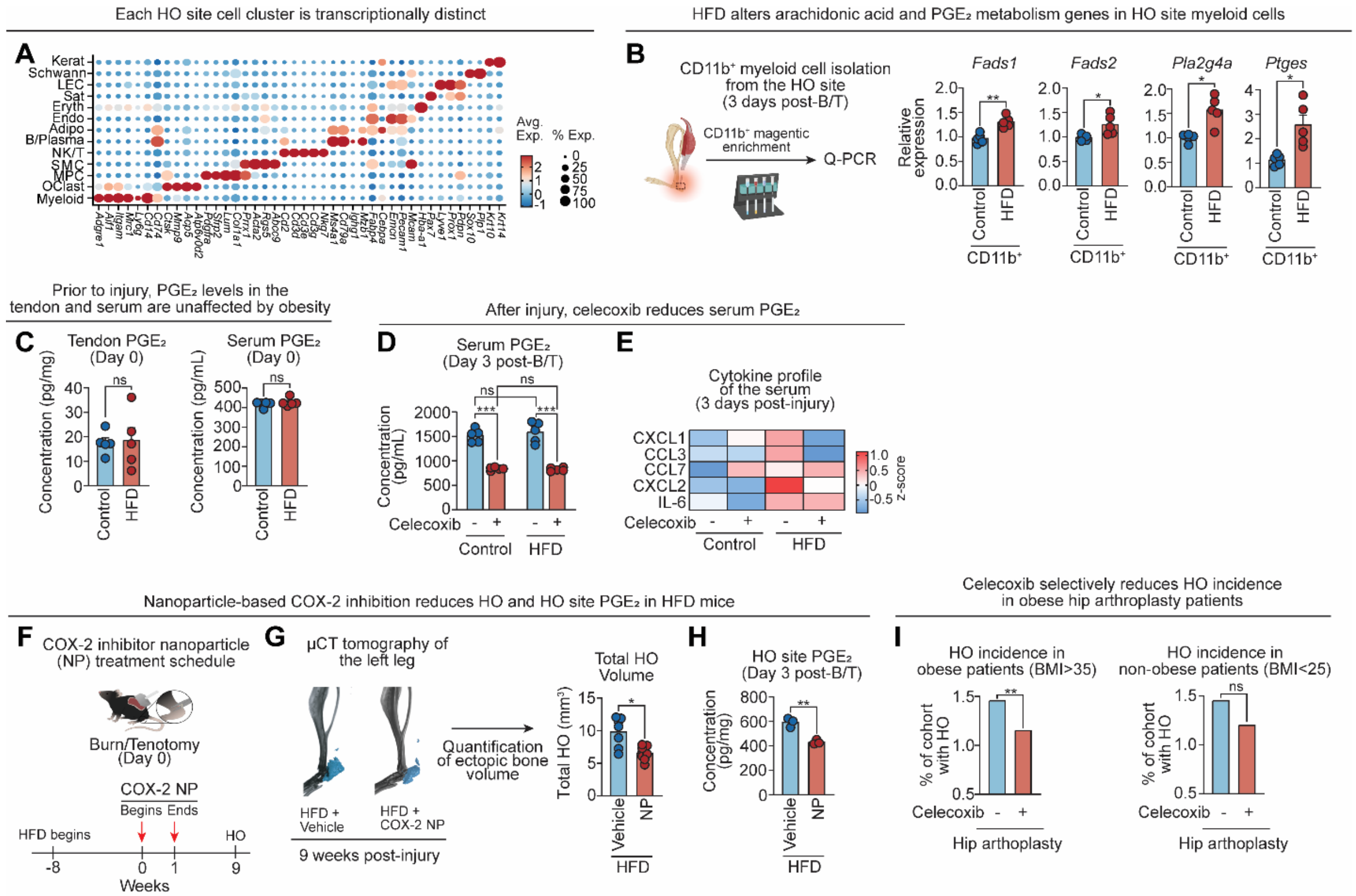


**Supplemental Figure 5: Myeloid COX-2 drives $PGE_2$ production at the HO site, which is suppressed by COX-2 inhibition.**

**(A)** Dot plot of canonical marker genes in each cluster from the scRNA-seq data obtained from control and HFD HO sites pre- and post-injury. Unbiased clustering categorized cells into the following cell types: B/plasma cells (n = 18,051 cells), myeloid cells (n = 64,110 cells), endothelial cells (n = 21,625 cells), mesenchymal progenitor cells (n = 46,547 cells), NK/T cells (n = 3,768 cells), satellite cells (n = 1,987 cells), smooth muscle cells (n = 8,475 cells), lymphatic endothelial cells (n = 1,207 cells), Schwann cells (n = 2,185 cells), adipocytes (n = 718 cells), erythrocytes (n = 581 cells), osteoclasts (n = 4,981 cells), and keratinocytes (n = 8,104 cells).

**(B)** Left: Schematic of $CD11b^+$ myeloid cell magnetic enrichment in control and HFD HO sites at day 3 post-B/T. Right: Bar graphs show the relative expression of genes related to LA/AA and $PGE_2$ metabolism (*Fads1, Fads2, Pla2g4a, and Ptges*) in $CD11b^+$ HO-site myeloid cells measured via qPCR (n = 5 mice/group).

**(C)** Quantification of tendon and serum $PGE_2$ at day 0 post-B/T. Tendon values are normalized to total protein (n = 5 mice/group).

**(D)** Quantification of serum $PGE_2$ in control, control + celecoxib, HFD, and HFD + celecoxib groups at day 3 post-B/T (n = 5 mice/group). Control and HFD values from Figure 4D are replotted to allow direct comparison with celecoxib-treated groups.

**(E)** Serum cytokines in control, control + celecoxib, HFD, and HFD + celecoxib mice at day 3 post-B/T. Values are normalized as z-scores (n = 4-5 mice/group).

**(F)** COX-2 inhibitor nanoparticle (COX-2 NP) treatment schedule. Dextran or dextran-conjugated COX-2 inhibitor-loaded nanoparticles were injected subcutaneously near the injury site daily for 1-week post-B/T in HFD mice.

**(G)** Representative µCT images of HO (left) and µCT quantification of total HO volume (right) at the ankle joint in each group (HFD + vehicle and HFD + COX-2 NP) at 9 weeks post- B/T (n = 7-8 mice/group).

**(H)** HO-site $PGE_2$ levels in HFD + vehicle and HFD + COX-2 NP-treated mice at day 3 post-B/T. Values are normalized to total protein (n = 3 mice/group).
**(I)** Incidence of HO in obese patients after hip arthroplasty treated with or without celecoxib (Left) and non-obese patients treated with or without celecoxib (Right) for 1-month post-surgery. (Left: n = 24,915 patients/cohort; Right: n = 11,191 patients/cohort).
HO, heterotopic ossification; B/T, burn/tenotomy; LA, linoleic acid; AA, arachidonic acid; COX, Cyclooxygenase; $PGE_2$, prostaglandin $E_2$; UMAP, uniform manifold approximation and projection; scRNA-seq, single-cell RNA-sequencing; NP, nanoparticle. Means ± SEM are plotted. All experiments are representative of at least two independent experiments. Statistics: Mann-Whitney (B, C, G), one-way ANOVA (D), two-tailed Student's t test (H), or Chi-Square (I). $*p < 0.05$; $**p < 0.01$; $****p < 0.0001$; ns, not significant.

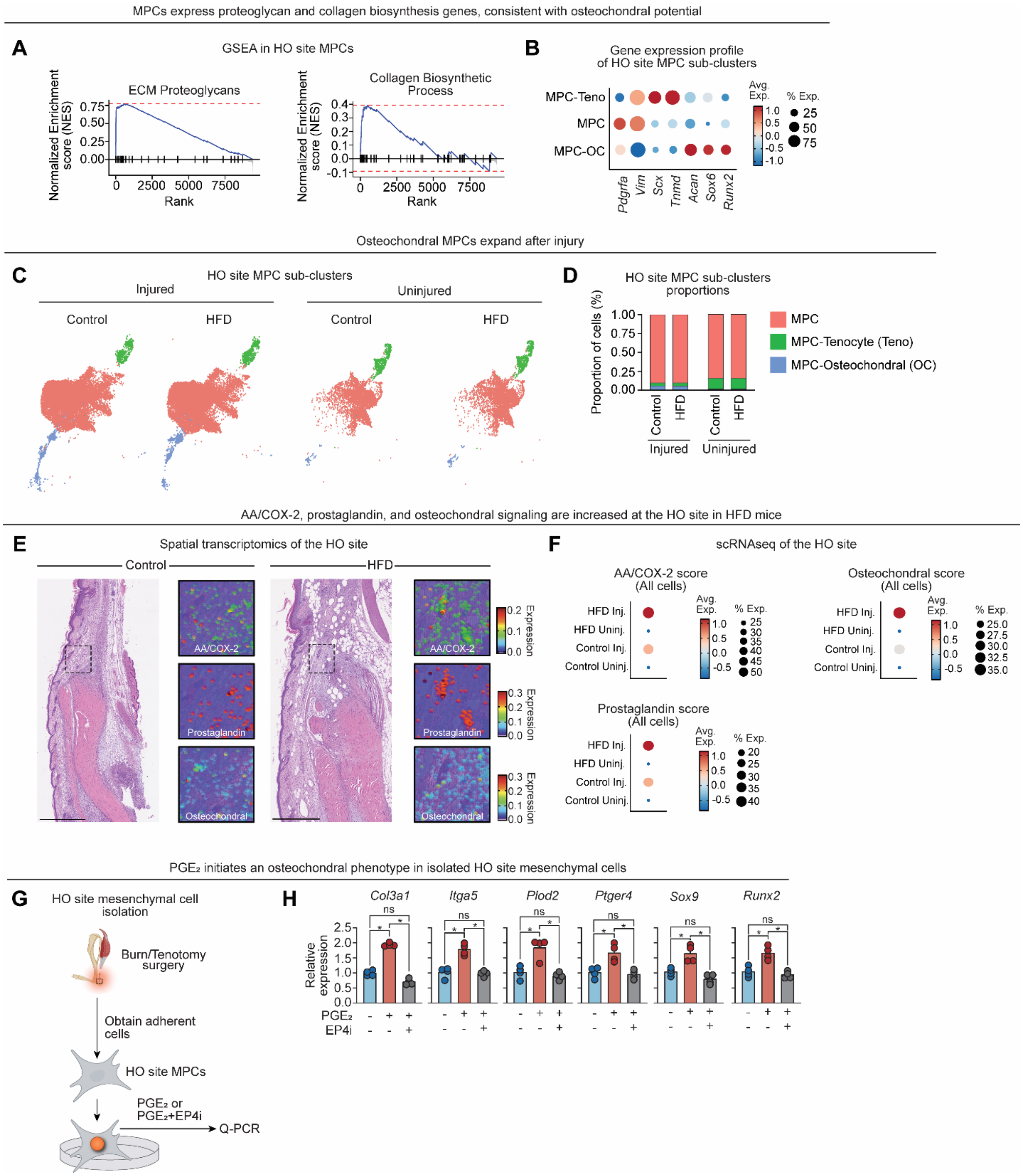


**Supplemental Figure 6: HFD-induced obesity and COX-2 signaling promote extracellular matrix and osteochondral gene programs at the HO site.**

**(A)** Gene set enrichment analysis (GSEA) using normalized enrichment scores (NES) of HFD vs. control MPCs. NES shows upregulation of ECM-proteoglycans and collagen biosynthesis in HFD MPCs relative to control MPCs after injury; Collagen Biosynthesis NES = 1.853, $p = 6.59 \times 10^{-3}$; ECM Proteoglycans NES = 3.361, $p = 1.019 \times 10^{-12}$.

**(B)** Gene expression profiles of the 3 HO site MPC subclusters (MPC-Teno, MPC-OC, and undifferentiated MPCs) defined by marker gene expression pre- and post-injury.
**(C)** UMAP of MPC subclusters, including MPC-Tenocytes (MPC-Teno) and MPC-Osteochondral cells (MPC-OC) split by injury status and diet.
**(D)** HO-site MPC sub-cluster proportions split by injury status and diet.
**(E)** Representative HD spatial gene expression of AA/COX-2-related, prostaglandin synthesis, and osteochondral differentiation genes within the HO-forming region at the distal end of the Achilles tendon 7 days post-injury. Each dot represents a cell with the color indicating its relative gene expression. (AA/COX-2-related genes: *Ptgs2*, *Ptger2*, *Ptger4*, *Fads1*, *Fads2*, *Ptges*, *Pla2g4a*, *Elovl2*, *Elovl5;* Prostaglandin synthesis genes*: Ptgs1, Ptgs2, Ptges;* Osteochondral differentiation genes: *Acan, Sox9, Ibsp, Col2a1, Bmp2, Bmp7, Runx2, Pdgfra*). Scale bar = 1mm.
**(F)** HO-site scRNA-seq analysis of corresponding gene module expression (AA/COX-2, prostaglandin synthesis, and osteochondral differentiation) from all cells pre- and post-injury in HFD and control mice.
**(G)** Schematic of MPC isolation and treatment with $PGE_2$. MPCs were isolated 7 days after B/T and treated with either $PGE_2$, $PGE_2$ + EP4 inhibitor (L-161,982), or vehicle for 24 hours, and subsequently collected for qPCR analysis.
**(H)** qPCR analysis of MPC osteochondral-related genes (*Col3a1, Itga5, Plod2, Ptger4, Sox9, Runx2*) treated with $PGE_2$, $PGE_2$ + EP4 inhibitor, or vehicle for 24 hours; n = 5 mice per group.
UMAP, uniform manifold approximation and projection; scRNA-seq, single-cell RNA-sequencing; MPC, mesenchymal progenitor cell; AA, arachidonic acid; COX-2, cyclooxygenase 2; $PGE_2$, prostaglandin $E_2$; EP4, Prostaglandin $E_2$ receptor 4. Means ± SEM are plotted. Statistics: one-way ANOVA (H). *$p < 0.05$; ns, not significant.

| Diet Name | Control | | HFD | | WD | | Low-LA HFD | |
|---|---|---|---|---|---|---|---|---|
| **Label** | gm | kcal | gm | kcal | gm | kcal | gm | kcal |
| Protein | 19.0 | 20.0 | 26.2 | 20.0 | 24.0 | 20.0 | 26.0 | 20.0 |
| Carbohydrate | 67.0 | 70.0 | 26.3 | 20.0 | 41.0 | 35.0 | 26.0 | 20.0 |
| Fat | 4.0 | 10.0 | 34.9 | 60.0 | 24.0 | 45.0 | 35.0 | 60.0 |
| Total | | 100.0 | | 100.0 | | 100.0 | | 100.0 |
| kcal/gm | 3.8 | | 5.2 | | 4.7 | | 5.2 | |
| **Ingredients** | **Control** | | **HFD** | | **WD** | | **Low-LA HFD** | |
| Added Cholesterol (mg) | 0.0 | 0.0 | 0.0 | 0.0 | 242.2 | 0.0 | 0.0 | 0.0 |
| Beef Tallow | 0.0 | 0.0 | 0.0 | 0.0 | 0.0 | 0.0 | 135.0 | 1215.0 |
| Calcium Carbonate | 5.5 | 0.0 | 5.5 | 0.0 | 5.5 | 0.0 | 5.5 | 0.0 |
| Casein | 0.0 | 0.0 | 200.0 | 800.0 | 200.0 | 800.0 | 200.0 | 800.0 |
| Casein, 30 Mesh | 200.0 | 800.0 | 0.0 | 0.0 | 0.0 | 0.0 | 0.0 | 0.0 |
| Cellulose | 0.0 | 0.0 | 0.0 | 0.0 | 50.0 | 0.0 | 0.0 | 0.0 |
| Cellulose, BW200 | 0.0 | 0.0 | 50.0 | 0.0 | 0.0 | 0.0 | 50.0 | 0.0 |
| Cholesterol | 0.0 | 0.0 | 0.0 | 0.0 | 0.1 | 0.0 | 0.0 | 0.0 |
| Cholesterol (mg/kg) | 0.0 | 0.0 | 0.0 | 0.0 | 282.2 | 0.0 | 0.0 | 0.0 |
| Choline Bitartrate | 2.0 | 0.0 | 2.0 | 0.0 | 2.0 | 0.0 | 2.0 | 0.0 |
| Coconut Oil | 0.0 | 0.0 | 0.0 | 0.0 | 0.0 | 0.0 | 0.0 | 0.0 |
| Coconut Oil, 101 | 0.0 | 0.0 | 0.0 | 0.0 | 0.0 | 0.0 | 0.0 | 0.0 |
| Corn Starch | 506.2 | 2024.8 | 0.0 | 0.0 | 0.0 | 0.0 | 0.0 | 0.0 |
| DiCalcium Phosphate | 13.0 | 0.0 | 13.0 | 0.0 | 13.0 | 0.0 | 13.0 | 0.0 |
| FD&C Blue Dye #1 | 0.0 | 0.0 | 0.1 | 0.0 | 0.0 | 0.0 | 0.0 | 0.0 |
| FD&C Red Dye #40 | 0.0 | 0.0 | 0.0 | 0.0 | 0.0 | 0.0 | 0.0 | 0.0 |
| FD&C Yellow Dye #5 | 0.0 | 0.0 | 0.0 | 0.0 | 0.1 | 0.0 | 0.0 | 0.0 |
| L-Cystine | 3.0 | 12.0 | 3.0 | 12.0 | 3.0 | 12.0 | 3.0 | 12.0 |
| Lard | 20.0 | 180.0 | 245.0 | 2205.0 | 177.5 | 1598.0 | 0.0 | 0.0 |
| Maltodextrin 10 | 125.0 | 500.0 | 125.0 | 500.0 | 0.0 | 0.0 | 125.0 | 500.0 |
| Menhaden Oil | 0.0 | 0.0 | 0.0 | 0.0 | 0.0 | 0.0 | 0.0 | 0.0 |
| Mineral Mix S10026 | 10.0 | 0.0 | 0.0 | 0.0 | 10.0 | 0.0 | 10.0 | 0.0 |
| Mineral Mix, S10026 | 0.0 | 0.0 | 10.0 | 0.0 | 0.0 | 0.0 | 0.0 | 0.0 |
| Olive Oil | 0.0 | 0.0 | 0.0 | 0.0 | 0.0 | 0.0 | 54.0 | 486.0 |
| Palm Oil | 0.0 | 0.0 | 0.0 | 0.0 | 0.0 | 0.0 | 81.0 | 729.0 |
| Potassium Citrate, 1 H2O | 16.5 | 0.0 | 16.5 | 0.0 | 16.5 | 0.0 | 16.5 | 0.0 |
| Safflower Oil | 0.0 | 0.0 | 0.0 | 0.0 | 0.0 | 0.0 | 0.0 | 0.0 |
| Soybean Oil | 25.0 | 225.0 | 25.0 | 225.0 | 25.0 | 225.0 | 0.0 | 0.0 |
| Sucrose | 68.8 | 275.2 | 68.8 | 275.0 | 345.6 | 1382.0 | 68.8 | 275.0 |
| Vitamin Mix V10001 | 10.0 | 40.0 | 10.0 | 40.0 | 10.0 | 40.0 | 10.0 | 40.0 |
| Total | 1055.05 | 4057.0 | 773.9 | 4057.0 | 858.223 | 4057.0 | 773.8 | 4057.0 |

**Supplemental Table 1: Dietary Ingredients**. Diet composition (grams) and caloric content (kcal) of rodent high-fat and control diets included in the study. HFD, high-fat diet; WD, Western diet; Low-LA HFD, low linoleic acid high-fat diet.

| Rank | Pathway | Impact | Scaled p-value | Sum |
|---|---|---|---|---|
| 1 | Linoleic acid metabolism | 1.00 | 0.64 | 1.64 |
| 2 | Taurine and hypotaurine metabolism | 0.60 | 0.85 | 1.45 |
| 3 | Arachidonic acid metabolism | 0.30 | 0.89 | 1.19 |
| 4 | Ascorbate and aldarate metabolism | 0.16 | 1.00 | 1.16 |
| 5 | Phenylalanine and tyrosine metabolism | 1.00 | 0.02 | 1.02 |

**Supplemental Table 2: HO-site Metabolic Pathway Analysis.** List of metabolic pathways elevated in HFD versus control HO sites, ranked by a composite score calculated as the sum of pathway impact (0-1) and scaled $-\log_{10}(p \text{ value})$ ($p$ values were normalized to 0-1 range).

| Fatty Acid Composition | | | | |
|---|---|---|---|---|
| | **Control** | **HFD** | **WD** | **Low-LA** |
| **ALA (g)** | 2.1 | 5.1 | 4.2 | 1.9 |
| **DHA (g)** | 0.0 | 0.0 | 0.0 | 0.0 |
| **EPA (g)** | 0.0 | 0.0 | 0.0 | 0.0 |
| **LA (g)** | 17.8 | 72.7 | 56.2 | 18.3 |
| **MUFA (% of total FA)** | 29.7 | 35.9 | 35.5 | 45.7 |
| **PUFA (% of total FA)** | 46.8 | 31.9 | 32.9 | 7.6 |
| **SFA (% of total FA)** | 23.5 | 32.2 | 31.6 | 43.5 |
| **Total MUFA (g)** | 12.8 | 91.2 | 67.7 | 122.0 |
| **Total PUFA (g)** | 20.2 | 81.0 | 62.8 | 20.4 |
| **Total SFA (g)** | 10.1 | 81.7 | 60.2 | 115.9 |
| **Total Fat (g)** | 43.1 | 253.9 | 190.7 | 258.3 |
| **kcal/g** | 3.8 | 5.2 | 4.7 | 5.2 |
| **ω-3 (g)** | 2.1 | 5.3 | 4.4 | 1.9 |
| **ω-6 (g)** | 17.9 | 73.7 | 57.0 | 18.4 |
| **ω-6/ω-3** | 8.4 | 13.9 | 13.1 | 9.8 |

**Supplemental Table 3: Fatty Acid Composition of all Diets.** Fatty acid composition (grams) of rodent high-fat and control diets included in the study. HFD, high-fat diet; WD, Western diet; Low-LA HFD, low linoleic acid high-fat diet; ALA, α linolenic acid; DHA, Docosahexaenoic acid; EPA, Eicosapentaenoic acid; LA, linoleic acid; SFA, Saturated fatty acids; MUFA, Monounsaturated fatty acids; PUFA, Polyunsaturated fatty acids; ω-3, omega-3; ω-6, omega-6.

**Supplemental Video 1 (related to Fig. S1): BlackBox recording of injured control diet mouse post-hip arthroplasty.** Representative video recording acquired from BlackBox from an injured control mouse 1 week following hip arthroplasty. This recording was used to calculate the total and standing hindpaw pressure index (left/right), which was quantified in Supplemental Figure 1, J-K.

**Supplemental Video 2 (related to Fig. S1): BlackBox recording of injured HFD mouse post-hip arthroplasty.** Representative video recording acquired from BlackBox from an injured HFD mouse 1 week following hip arthroplasty. This recording was used to calculate the total and standing hindpaw pressure index (left/right), which was quantified in Supplemental Figure 1, J-K.

**Supplemental Video 3 (related to Fig. S1): BlackBox recording of uninjured control diet mouse.** Representative video recording acquired from BlackBox from an uninjured control mouse. This recording was used to calculate the total and standing hindpaw pressure index (left/right), which was quantified in Supplemental Figure 1, J-K.

**Supplemental Video 4 (related to Fig. S1): BlackBox recording of uninjured HFD diet mouse.** Representative video recording acquired from BlackBox from an uninjured HFD mouse. This recording was used to calculate the total and standing hindpaw pressure index (left/right), which was quantified in Supplemental Figure 1, J-K.
